# Environmental predictors of *Escherichia coli* concentration at marine beaches in Vancouver, Canada: a Bayesian mixed-effects modelling analysis

**DOI:** 10.1017/S0950268824000311

**Published:** 2024-02-26

**Authors:** Binyam N. Desta, Jordan Tustin, J. Johanna Sanchez, Cole Heasley, Michael Schwandt, Farida Bishay, Bobby Chan, Andjela Knezevic-Stevanovic, Randall Ash, David Jantzen, Ian Young

**Affiliations:** 1 School of Occupational and Public Health, Toronto Metropolitan University, Toronto, ON, Canada; 2 Vancouver Coastal Health, Vancouver, BC, Canada; 3 School of Population and Public Health, University of British Columbia, Vancouver, BC, Canada; 4 Metro Vancouver, Vancouver, BC, Canada

**Keywords:** Bayesian, environmental factors, *Escherichia coli* (*E. coli*), marine water, Vancouver

## Abstract

Understanding historical environmental determinants associated with the risk of elevated marine water contamination could enhance monitoring marine beaches in a Canadian setting, which can also inform predictive marine water quality models and ongoing climate change preparedness efforts. This study aimed to assess the combination of environmental factors that best predicts *Escherichia coli* (*E. coli)* concentration at public beaches in Metro Vancouver, British Columbia, by combining the region’s microbial water quality data and publicly available environmental data from 2013 to 2021. We developed a Bayesian log-normal mixed-effects regression model to evaluate predictors of geometric *E. coli* concentrations at 15 beaches in the Metro Vancouver Region. We identified that higher levels of geometric mean *E. coli* levels were predicted by higher previous sample day *E. coli* concentrations, higher rainfall in the preceding 48 h, and higher 24-h average air temperature at the median or higher levels of the 24-h mean ultraviolet (UV) index. In contrast, higher levels of mean salinity were predicted to result in lower levels of *E. coli.* Finally, we determined that the average effects of the predictors varied highly by beach. Our findings could form the basis for building real-time predictive marine water quality models to enable more timely beach management decision-making.

## Key results

- Higher geometric mean *E. coli* levels were predicted by higher previous sample day *E. coli* concentrations, higher rainfall in the preceding 48h, and higher 24-h average air temperature at the median or higher levels of the 24-h mean UV index
- Higher levels of mean salinity were predicted to result in lower levels of geometric *E. coli*
- The average effects of each predictor on the *E. coli* count varied highly by beaches, which indicates that a beach-specific approach to beach monitoring programmes and predictive models is warranted in Metro Vancouver

## Introduction

Swimming in marine beaches can cause acute gastrointestinal, respiratory, and other illnesses due to exposure to enteric pathogens [1]. Faecal contamination of such water bodies can also pose aesthetic concerns to beachgoers, diminish beach usage and related health benefits [2], and result in economic losses due to beach closures upon detecting such pollution [3]. Routine monitoring of recreational beaches for faecal indicator bacteria (FIBs) allows public health authorities to issue notifications or beach closures when the water is unsafe for swimming and when health risk threshold levels are exceeded [4]. In Canada, *Enterococci* are the recommended FIBs for marine beach waters, with *Escherichia coli* (*E. coli*) primarily used for freshwater sources [5]. However, *E. coli* can also be used in marine water if they are found to demonstrate faecal contamination in such waters [6]. Both FIBs are associated with faecal contamination and are often used interchangeably as a predictor of the risk of gastrointestinal illness among beachgoers [7]. Previously, the Canadian recreational water guideline set a health threshold for gastrointestinal illness as a geometric mean > 200 colony-forming unit (CFU)/100 ml or a single sample > 400 CFU/100 ml of *E. coli*; the updated 2023 guidelines now specify that any single sample > 235 CFU/100 ml should result in public health follow-up actions for possible increases in health risks [6, 8].

Detecting FIB using culture-based laboratory procedures takes 18–24 h, meaning public notifications, if any, would happen the next day after sampling. However, complex processes and hydro-meteorological conditions affect the coastal water quality, and FIB’s concentration can vary over time scales from minutes to days [9]. In Canada, most local and provincial health authorities still use culture-based methods as the primary detection method, though rapid polymerase chain reaction (PCR)-based techniques are now recommended for wider adoption [6]. Thus, existing beach water monitoring and reporting programmes cannot accurately identify real-time unsafe swimming conditions and consequent health risks [10].

Higher risks of faecal contamination of recreational waters are associated with various environmental determinants [11]. Recent rainfall has been consistently reported as a predictor of marine recreational water quality [12, 13]. Salinity also influences the survival of FIB in marine water bodies [14]. The varying effect of temperature and ultraviolet (UV) radiation on FIB in marine water has also been indicated [14–16]. In the future, with the ongoing climate change impacts in place, these parameters can fluctuate widely, thereby altering the concentration of FIB in marine water and the consequent risk of developing recreational water illness [17].

The complexity of how these conditions affect marine water quality urges identifying their region- and beach-specific impacts [9]. Such environmental determinants associated with higher risks of marine water contamination, if identified and monitored, would serve as a critical component of enhancing the monitoring of recreational water quality. No studies have been conducted on identifying the environmental predictors of *E. coli* concentration in marine recreational water in a Canadian setting. Vancouver is Canada’s largest city located on marine water, with several popular beaches in and around the city. We aimed to assess the combination of environmental factors that best predicts *E. coli* concentration at public beaches in Metro Vancouver, British Columbia, by combining the region’s microbial water quality data and publicly available environmental data. These study findings will inform the province’s existing beach water management, predictive modelling, and climate change preparedness.

## Materials and methods

### Study area

**Figure 1.**
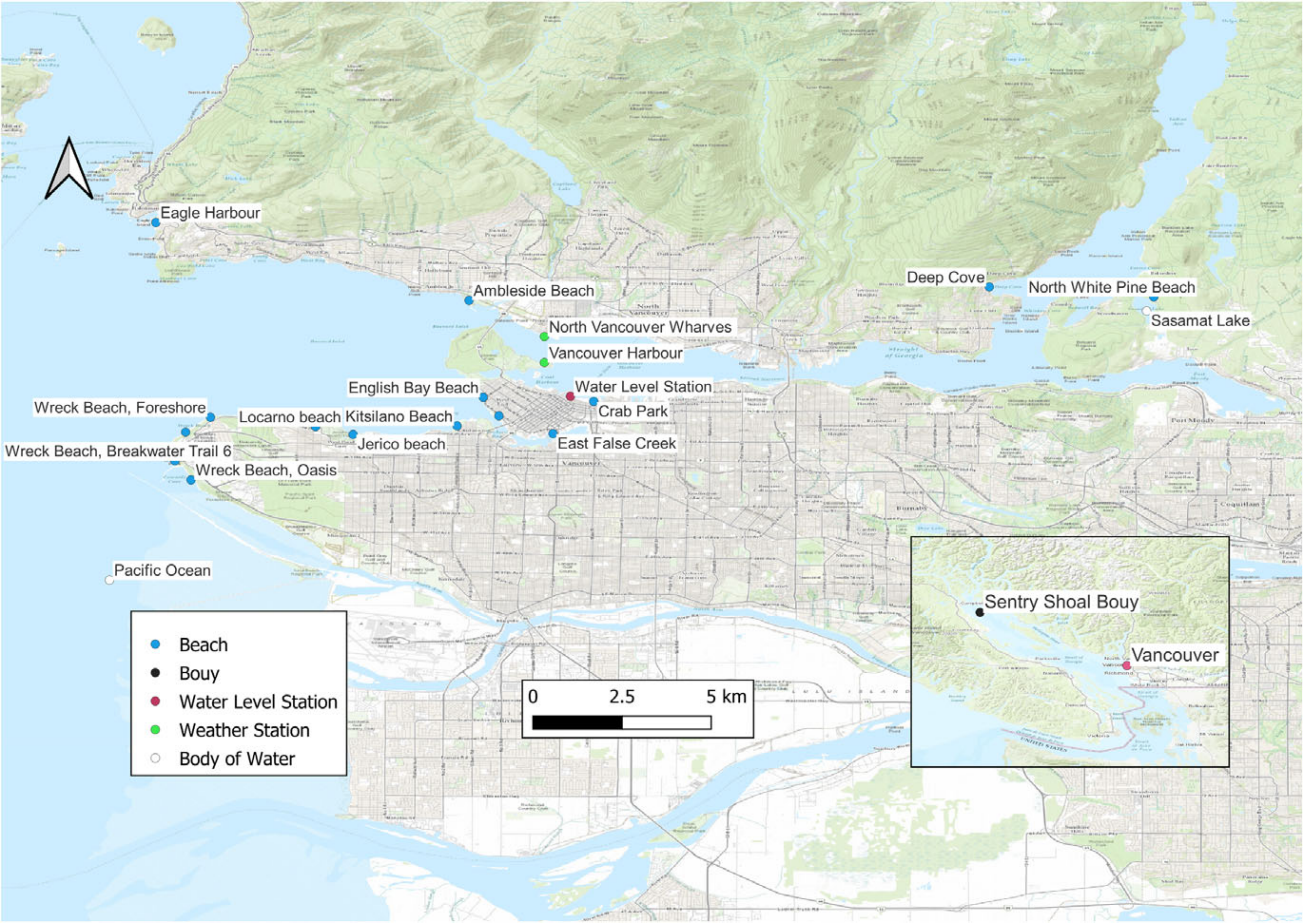
Selected beaches and environmental monitoring stations in the Metro Vancouver Region (2013–2021).

This study included 15 selected beaches monitored for recreational water quality in Metro Vancouver (Figure 1). These beaches were selected based on their popularity, elevated historical FIB counts, and known or suspected sources of faecal pollution. More than half of the people (>2.4 million) in the province of British Columbia live in Metro Vancouver, making it the 3rd and 31st largest metro area in Canada and North America, respectively [18]. Vancouver (with most of the beaches by the Pacific Ocean) is also one of Canada’s top tourist destinations, with an annual 9.2 million visitors. Notably, the proportions of visitors are more evenly distributed to all seasons compared to other tourism regions in Canada [19]. Vancouver’s beaches are famous for bathing (from open-water swimming to wading) and other non-bathing water activities among both local residents and tourists. Many of the beaches are an integral part of the well-used park network in the region, while some host popular annual festivals and others are popular nudist beaches. Some of the 15 recreational beaches we studied are not recognized by the local municipality as bathing beaches for swimming, but they are used for secondary contact activities (e.g. paddleboarding, dragon boating, and kayaking) that could still lead to water exposure.

### Data set description

We analysed a data set of routinely collected beach water quality data obtained from Metro Vancouver from 2012 to 2021. Metro Vancouver conducts routine microbial water quality monitoring of the region’s beaches weekly throughout the beach season between mid-April and the end of September. In addition, about one-third of the beaches are repeat sampled each week, where three routes were randomly assigned from Monday to Wednesday each week, and then, one of them was repeated on a Thursday to provide for at least five samples within the 30 days. Overall, each site at each beach location was sampled around 26 weeks plus its one-third or a total of 32–34 times per year. Depending on factors such as location, when the following schedule sample is planned, and weather, sometimes beaches were resampled when elevated counts were observed to confirm these results. English Bay Beaches, a location where Vancouver’s Annual Polar Bear swim takes place, were also monitored for about a month in December, and the geometric mean was calculated before the beginning of January every year.

In Vancouver, the FIB shifted from faecal coliforms to *E. coli* in 2013, and our analysis focused on data for *E. coli* only (2013–2021). Samples were analysed using the Colilert‐18® system and the Quanti‐Tray Most Probable Number (MPN) technique to measure the *E. coli* abundance [20]. They calculated a 30-day running geometric mean concentration for each beach weekly to determine health risk thresholds. Hence, a new geometric mean was calculated every time a beach was sampled. The local health authority, Vancouver Coastal Health (and Fraser Health, which monitored two of the fifteen beaches in this study), used the results to issue beach advisories (except for two beaches with no advisories) when the 30-day geometric *E. coli* concentration exceeded the health risk threshold concentration of 200 *E. coli* per 100 mL of water, as per the Health Canada guideline during the time period [8]. Local authorities also used a maximum threshold concentration of 400 *E. coli* bacteria per 100 ml of water to note short-term water quality concerns [8].

### Environmental data

At the time of water sampling, Metro Vancouver measured salinity as a unitless analyte until 2019, when the method was changed to specify a unit of parts per thousand (PPT). The region also started collecting water temperatures during water sample collection in 2015. However, since two years of water temperatures were unavailable and we needed to explore more predictors of *E. coli* concentrations on the beach, we used environmental data from other sources for this study. First, we gathered daily rainfall and air temperature data from two Environment and Climate Change Canada weather stations in average proximity to the beaches from 2013 to 2021 [21, 22]. We collected UV index data from a National Aeronautics and Space Administration (NASA, USA) solar and meteorological parameters database, with data starting from 2012 [23]. Finally, we obtained wind speed and direction, gust speed, wave height, and water surface temperature data (Sentry Shoal Buoy) [24] and water level data [25] from a Department of Oceans and Fisheries buoy, with data starting in 2012.

### Statistical analysis

We imported the merged data set into RStudio (R version 4.2.2) for preparation and analysis. Same-day values of total precipitation (in millimetres, mm), mean air temperature (in degree centigrade, °C), mean salinity (unitless and PPT), water level above a standard (in metres), average UV index (one UV index unit is equivalent to 25 milliwatts per square metre), and antecedent dry days (number of days since the last rainfall) were the environmental factors considered for this analysis. To choose variables to include in the model, we reviewed prior literature and developed and used a directed acyclic graph (DAG: Figure A in the Supplementary Material) [26]. Accordingly, we excluded water level from the final model for its mediator effect [27] in relation to rainfall and air temperature with *E. coli* concentration [28]. Even though the water sampling frequency was not daily, we explored the effect of *E. coli* geometric mean values from the previous sample day at each beach. We also assessed the previous-day values of mean air temperature and average UV index to evaluate temporality. For rainfall, we included a sum of values for the last two days from the day of water sample collection (i.e. 48-h cumulative rain in the previous two days). We filled in some missing temperature and rainfall values by merging data sets from two nearby weather stations (i.e. Vancouver Harbour [22] and North Vancouver Wharves [21]). We excluded missing data for specific variables from the analysis of that variable. We analysed data from 2013 to 2021 to include the most complete data on environmental factors. We treated each monitored beach as a separate beach in this analysis, regardless of geographical proximity.

We used a Bayesian framework to conduct the analysis, which estimates posterior distributions of model parameters to include uncertainty and allows direct probability statements based on model results [29]. We developed multilevel regression models to determine which combination of environmental predictors best predicts *E. coli* geometric mean levels at the Vancouver beaches. As a first step, we centred and standardized all potential predictor variables for easier comparisons, prior distribution specification, and interpretation in our complex model with interaction terms. Further, we log-transformed (natural) the *E. coli* previous sample day geometric mean variable to reduce skewness.

We started our multilevel regression modelling by examining the relationship between environmental predictors and a log-transformed *E. coli* geometric mean outcome using a Gaussian (normal) distribution. In this first model, we included the previous sample day log *E. coli* geometric mean, 48-h cumulative rainfall, 24-h mean air temperature, mean salinity, 24-h average UV index, antecedent dry days, and year as fixed effects and beach as a varying intercept parameter. We changed the distribution to log-normal to improve the model fit and ran the same model for the untransformed geometric mean *E. coli* outcome. To further enhance the model fit based on the posterior predictive checks, which assessed the appropriateness of the model to simulate new data for the observed data, we opted to fit a varying slope for each predictor variable for each beach. We also tested whether using the year as a cross-classified varying effect would improve model fit. However, given the increased model complexity, we found limited benefits; therefore, we kept the year as a fixed effect. Then, we assessed a two-way interaction effect of 24-h mean temperature and 24-h mean UV index on the *E. coli* outcome, informed by our DAG. We specified weakly informative priors for all model beta-coefficient parameters and varying effect correlations. The specified priors had normal distributions for the fixed-effect parameters with a mean of 0 and a standard deviation (SD) of 1. We selected a weakly informative Cholesky correlation factor Lewandowski–Kurowicka–Joe (LKJ(2)) prior for the varying slope effects. For SD parameters, we used a Student *t* distribution with three degrees of freedom, a mean of 0, and a sigma of 2.5.

We built the models using the ‘brms’ package (and ‘CmdStanR’ interface) in RStudio to fit the model by the use of Stan probabilistic programming software [30]. The models were estimated using Hamiltonian Monte Carlo sampling. We used 2,000 iterations across each of the four chains using four cores to estimate the models. We used trace plots, r-hat values, and effective sample sizes to assess model convergence. We used conditional adjusted prediction plots to verify (via visual detection of direct/indirect proportional association with the *E. coli* outcome) the appropriateness of varying slopes for each predictor (see Figures B–F in the Supplementary Material). We performed posterior predictive checks to evaluate the models’ suitability in simulating generated data with the observed data. We used expected log predictive density to compare the models using the ‘loo_compare’ function. We produced and plotted posterior predictions of the expected value of the final model parameters per each predictor using the ‘marginaleffects’ package. We also calculated the average marginal effects to visually evaluate the impact of each predictor variable on the *E. coli* counts. All the predictor variables were transformed back to their original scale for the marginal effects plots. In our final model output plots, we displayed the parameter distribution densities, median value, and 80% and 95% credible intervals (CIs). As a sensitivity analysis to check the impact of data missingness, we built a full Bayesian imputation model of missing data of our final model within ‘brms’ package. In the model, we combined the complete data likelihood with prior information to compute the complete data posterior, and we specified the variables that contain missing values [29, 31]. This study’s formatting and analysis of R script files and the data set are available from the following GitHub page: https://github.com/bndesta/VancouverRecWater.

## Results

### Descriptive data

We included 4,770 geometric mean *E. coli* observations collected between 2013 and 2021 across 15 recreational areas in the study. The summary statistics of all variables used in predicting *E. coli* outcomes at the beaches are presented in Table 1.

**Table 1.** Summary statistics of predictor variables of geometric mean *E. coli* concentration at 15 beaches in the Metro Vancouver Region, 2013–2021

Variables	Number	Mean	SD	Range
*E. coli* geometric mean (CFU/100 ml)	4,770	155.0	800.0	24,186.0
Previous sample day log *E. coli* geometric mean (CFU/100 ml)	4,755	3.4	1.4	7.8
48-h total rainfall (mm)	4,582	3.8	8.4	76.5
Mean salinity (PPT)	4,760	15.6	7.0	34.2
Antecedent dry days	4,777	3.9	5.4	31.0
24-h mean air temperature (°C)	4,727	16.4	4.1	30.3
24-h mean UV index	4,777	1.29	0.5	2.5

**Figure 2.**
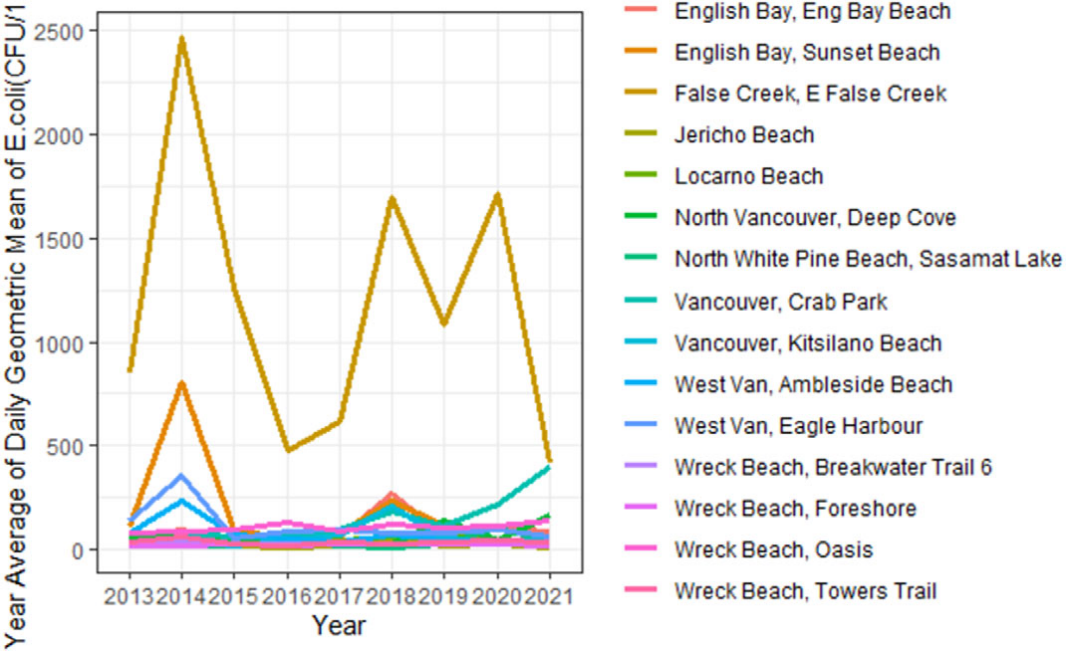
Mean annual geometric mean at beaches in the Metro Vancouver Region, 2013–2021.

The yearly average geometric mean of *E. coli* concentration varied by beach (Figure 2). The highest mean annual geometric *E. coli* concentration observed in all study years was at East False Creek (which is not recognized for bathing) (Figure 2).

### Bayesian mixed-effects regression model

Our final mixed-effects model included beach as a varying effect with varying slopes for each predictor, except for antecedent dry days, and a two-way interaction effect of 24-h mean temperature and 24-h mean UV index on the *E. coli* outcome. The model showed no issues with convergence (see Figures G and H in the Supplementary Material). We also checked the appropriateness of varying slopes for each predictor via visual detection of direct/indirect proportional association with *E. coli* outcome (see Figures B–F in the Supplementary Material). The leave-one-out cross-validation (LOO) model comparison indicated that our final model had better predictive performance than the model without the interaction effect and antecedent dry days variable as a varying slope (Tables A and B in the Supplementary Material).

**Figure 3.**
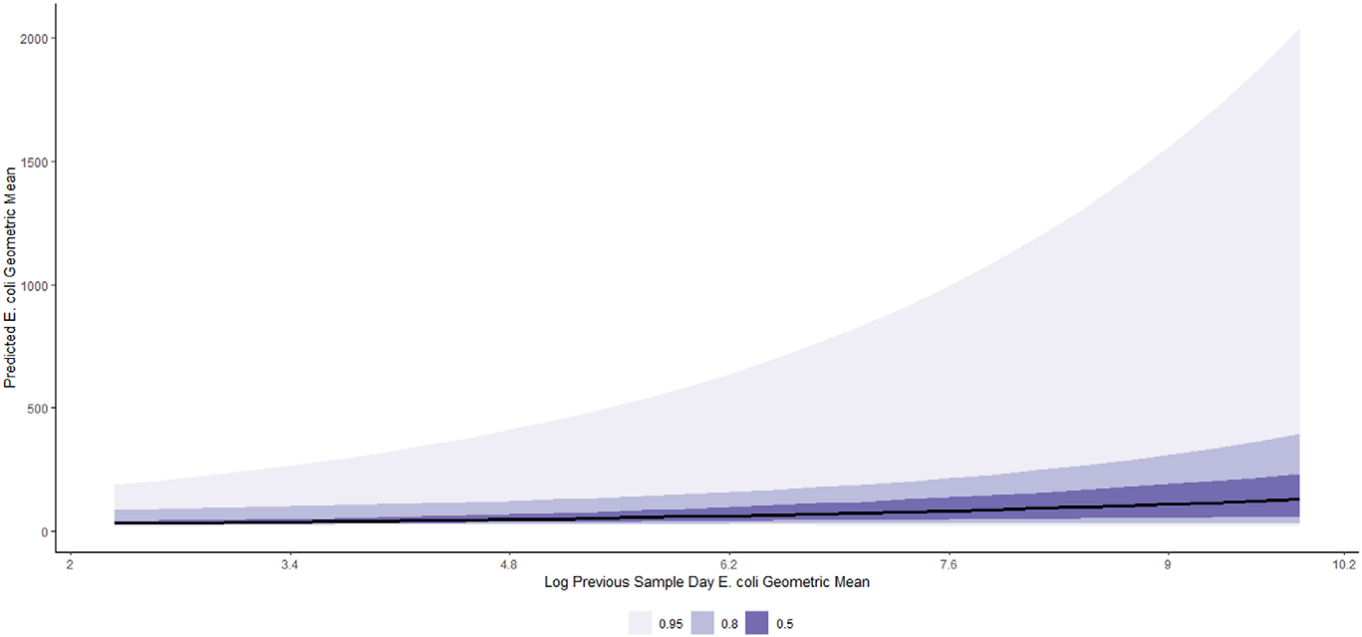
Posterior predictions of the average expected value of the geometric *E. coli* concentration per value of previous sample day log geometric mean of *E. coli* at beaches in the Metro Vancouver Region, 2013–2021.

**Figure 4.**
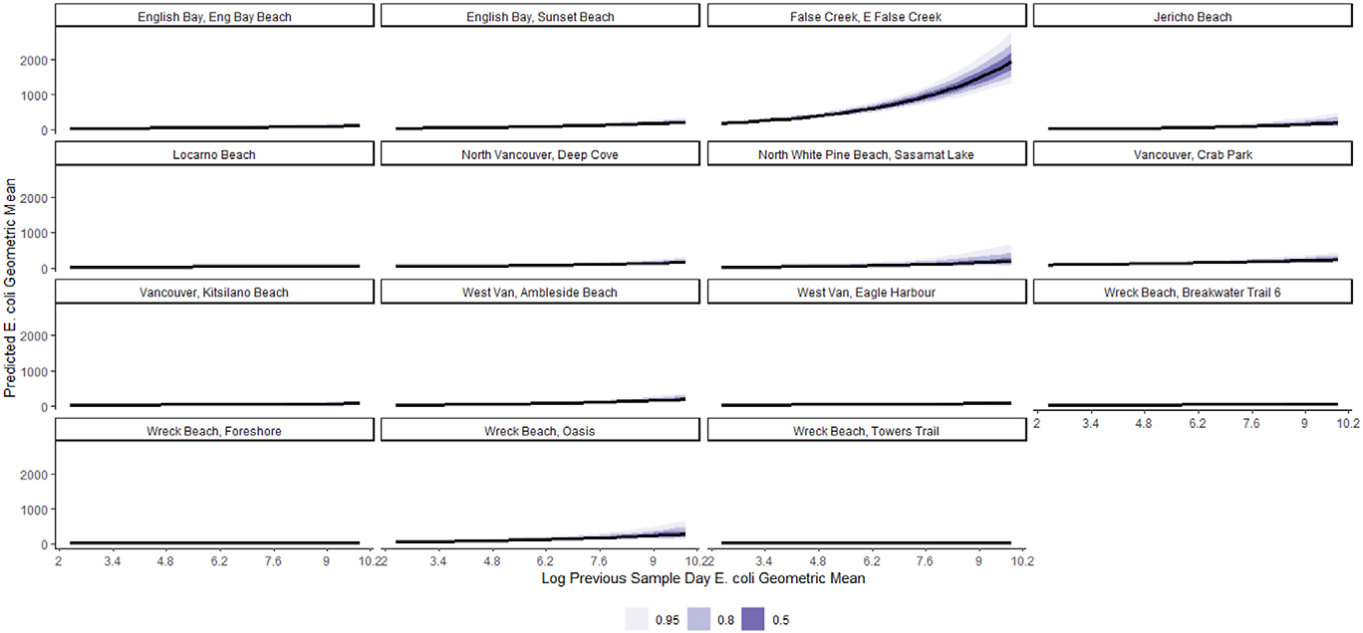
Posterior predictions of the beach-specific average expected value of the geometric *E. coli* concentration per value of previous sample day log geometric mean of *E. coli* at beaches in the Metro Vancouver Region, 2013–2021.

We present estimates of the log-normal mixed-effects regression model for geometric mean *E. coli* concentration in Table 2. The overall and beach-specific posterior predictions for the average expected value of the geometric mean *E. coli* concentration per value of each predictor variable are more intuitively shown in Figures. Conditional on other predictors in the model, on average, a 1-SD (1.4 log CFU/100 ml) increase in the previous sample day log geometric mean of *E. coli* was associated with a 23% (95% CI: 12%, 34%) increase in geometric mean *E. coli* counts (Table 2). This effect is visualized in Figure 3. Figure 4 shows a varying pattern by beaches, with the strongest effect of the previous sample day log geometric mean *E. coli* noted at East False Creek and no effect detected in Locarno and Wreck (Foreshore) beaches.

**Table 2.** Bayesian log-normal mixed-effects model of the relationship between environmental factors and geometric mean *E. coli* concentration at 15 beaches in the Metro Vancouver Region, 2013–2021 (other parameters, including the correlation effects of the varying slopes and the coefficients for year, are included in Table C in the Supplementary Material)

Outcome/parameter^a^	Estimate^b^	95% credible interval	R-hat^c^	Bulk ESS^c^	Tail ESS^c^
*Fixed-level effects*					
Intercept	3.17	(2.79, 3.54)	1.01	439	821
Previous sample day log *E. coli* geometric mean	0.23	(0.13, 0.34)	1.00	901	1,496
48-h total rainfall	0.20	(0.16, 0.24)	1.00	2,998	2,692
Mean salinity	−0.28	(−0.41, −0.13)	1.00	1,212	1,674
Antecedent dry days	0.01	(−0.03, 0.04)	1.00	4,860	2,965
2-h mean air temperature	0.31	(0.21, 0.41)	1.00	1,521	2,415
24-h mean UV	−0.13	(−0.22, −0.05)	1.00	1,450	2,167
24-h mean air temperature * 24-h mean UV (*interaction term*)	0.19	(0.11, 0.26)	1.00	2,167	2,443
*Group-level effects* for beach (SD)	1.02	(1.00, 1.04)	1.00	6,875	2,838

a Models conditioned on the study year as a fixed effect.

b All the fixed-effects estimates and credible intervals are shown here on the mean-centred and standardized scale.

c R-hat values indicate model convergence, with values closer to 1 indicating convergence. Bulk and tail effective sample size (ESS) are indicators of Markov chain sampling efficiency, with higher numbers showing more reliable results.

**Figure 5.**
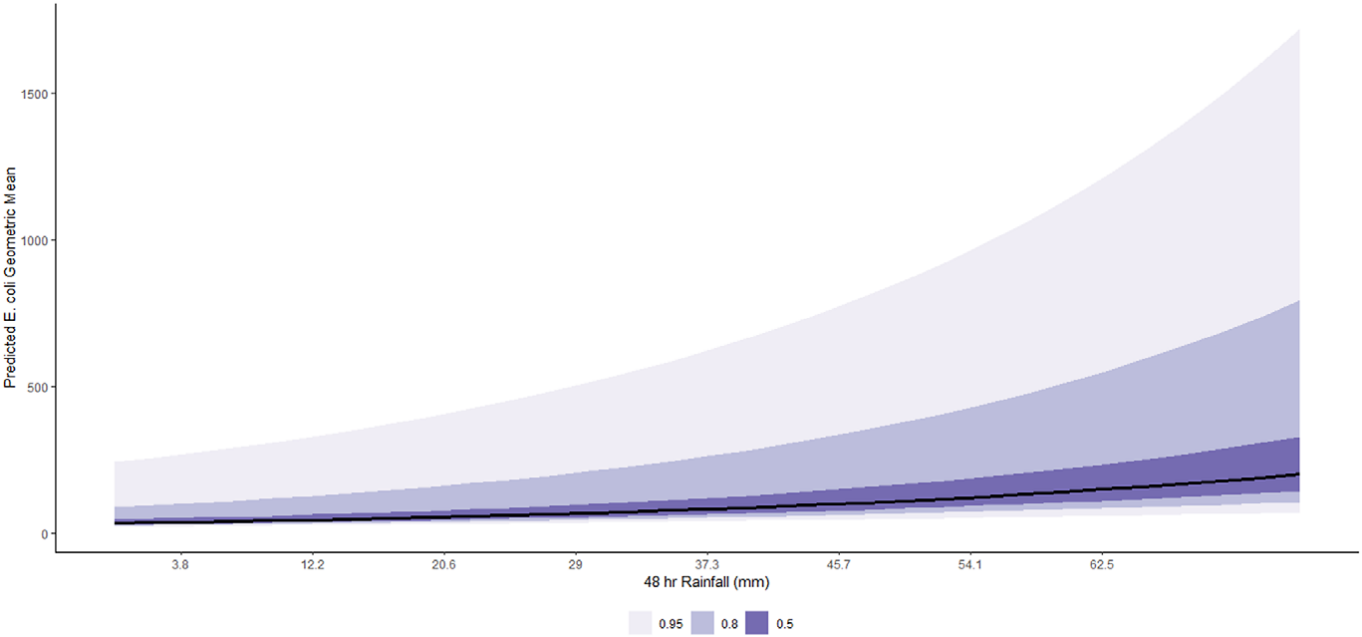
Posterior predictions of the average expected value of the geometric *E. coli* concentration per value of 48-h total rainfall at beaches in the Metro Vancouver Region, 2013–2021.

**Figure 6.**
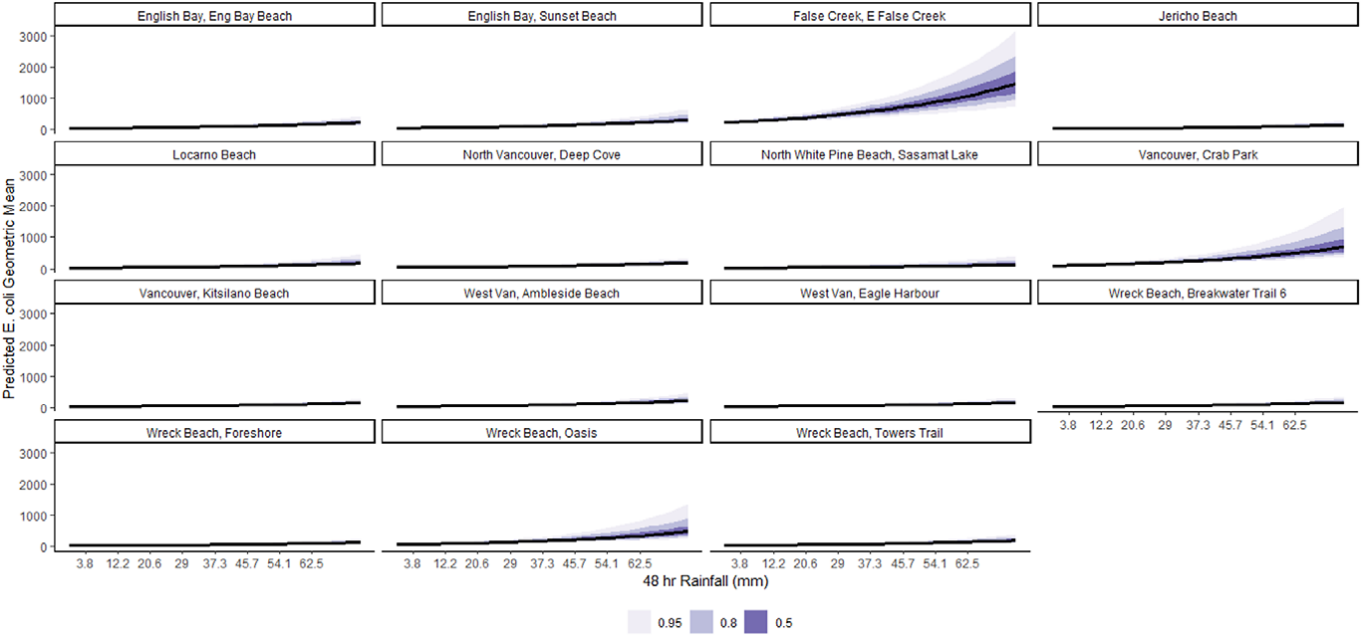
Posterior predictions of the beach-specific average expected value of the geometric *E. coli* concentration per value of 48-h total rainfall at beaches in the Metro Vancouver Region, 2013–2021.

**Figure 7.**
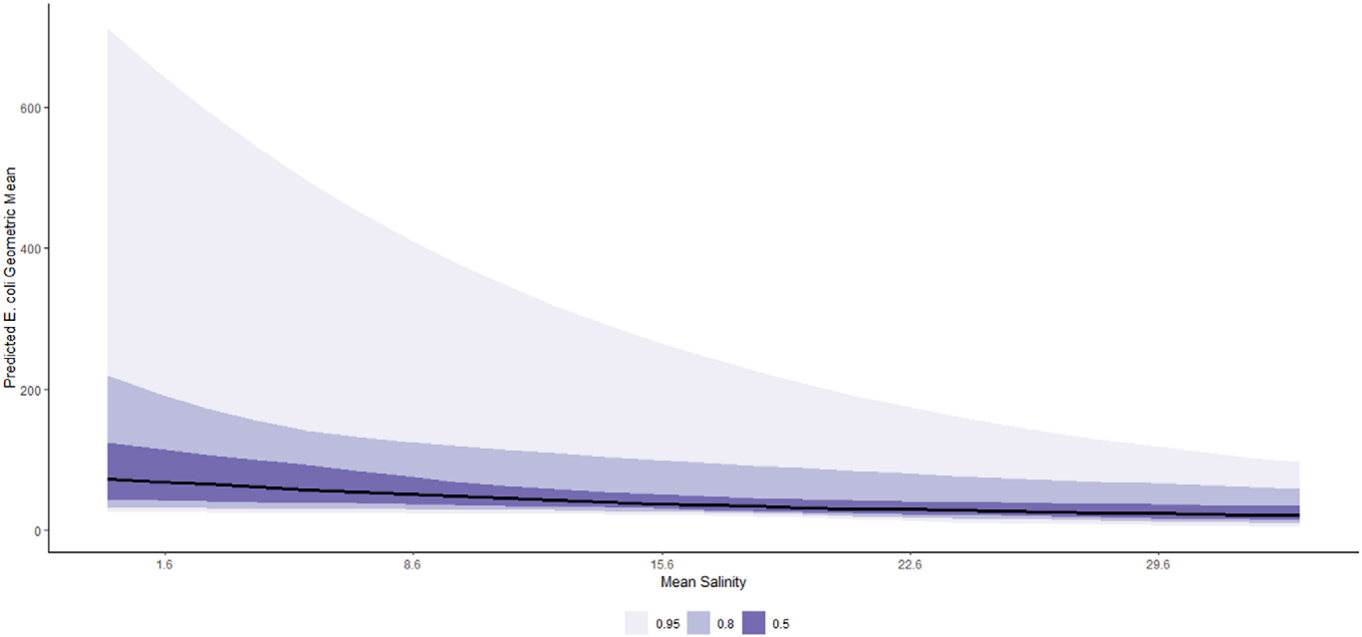
Posterior predictions of the average expected value of the geometric *E. coli* concentration per value of mean salinity at beaches in the Metro Vancouver Region, 2013–2021.

**Figure 8.**
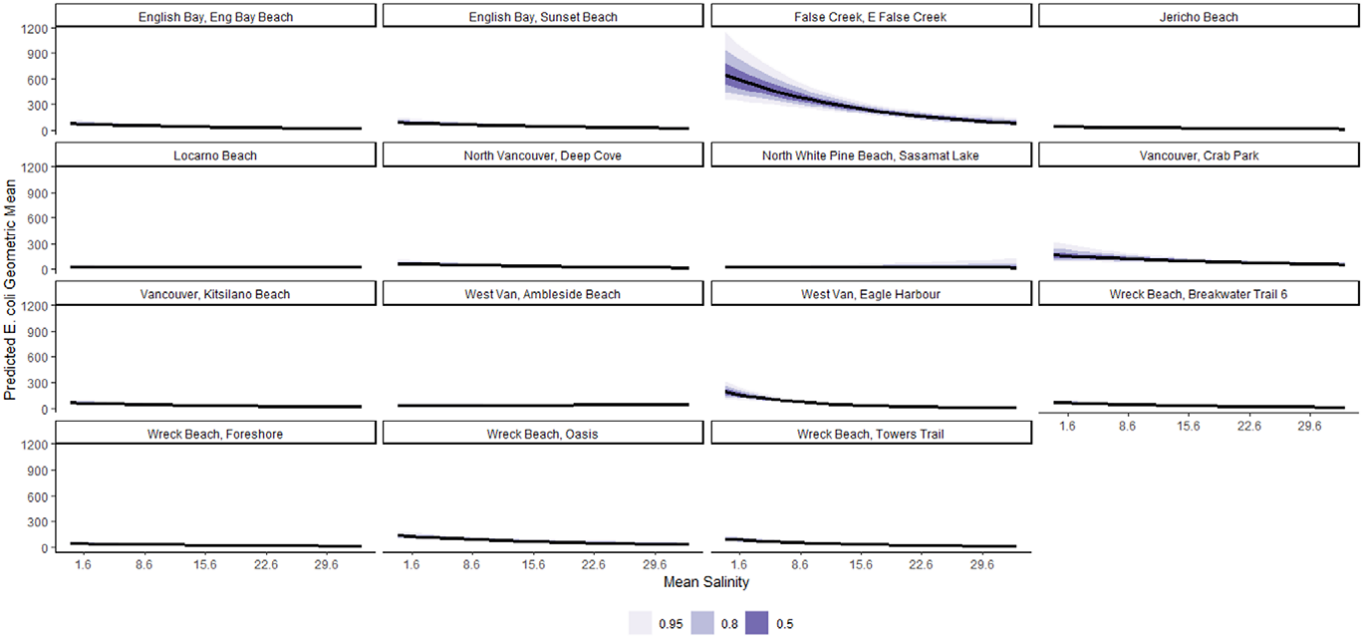
Posterior predictions of the beach-specific average expected value of the geometric *E. coli* concentration per value of mean salinity at beaches in the Metro Vancouver Region, 2013–2021.

On average, a 1-SD (8.4 mm) increase in 48-h cumulative rainfall was associated with a 20% (95% CI: 16%, 24%) increase in geometric mean *E. coli* concentration, conditional on other predictors in the model (Table 2). The marginal effects of this relationship are shown in Figure 5. This association is remarkably varied by beach (Figure 6), with the strongest relationships noted in East False Creek, Wreck Beach (Oasis), and Crab Park. Conditional on other predictors in the model, on average, a 1-SD (7 PPT) increase in mean salinity was associated with a 27% (95% CI: 13%, 41%) decrease in geometric mean *E. coli* counts (Table 1). The marginal effects are shown in Figure 7, which are also notably varied by beaches (Figure 8). For example, a decreasing trend was most strongly noted at East False Creek.

On average, antecedent dry days showed effectively no association with geometric mean *E. coli* counts (mean: 0.01; 95% CI: −0.03, 0.04) for a 1-SD (5.4 days) increase in antecedent dry days, conditional on other predictors in the model (Table 1). Figure I in the Supplementary Material illustrates this lack of association, where the variation occurred only at the initial (intercept) level.

**Figure 9.**
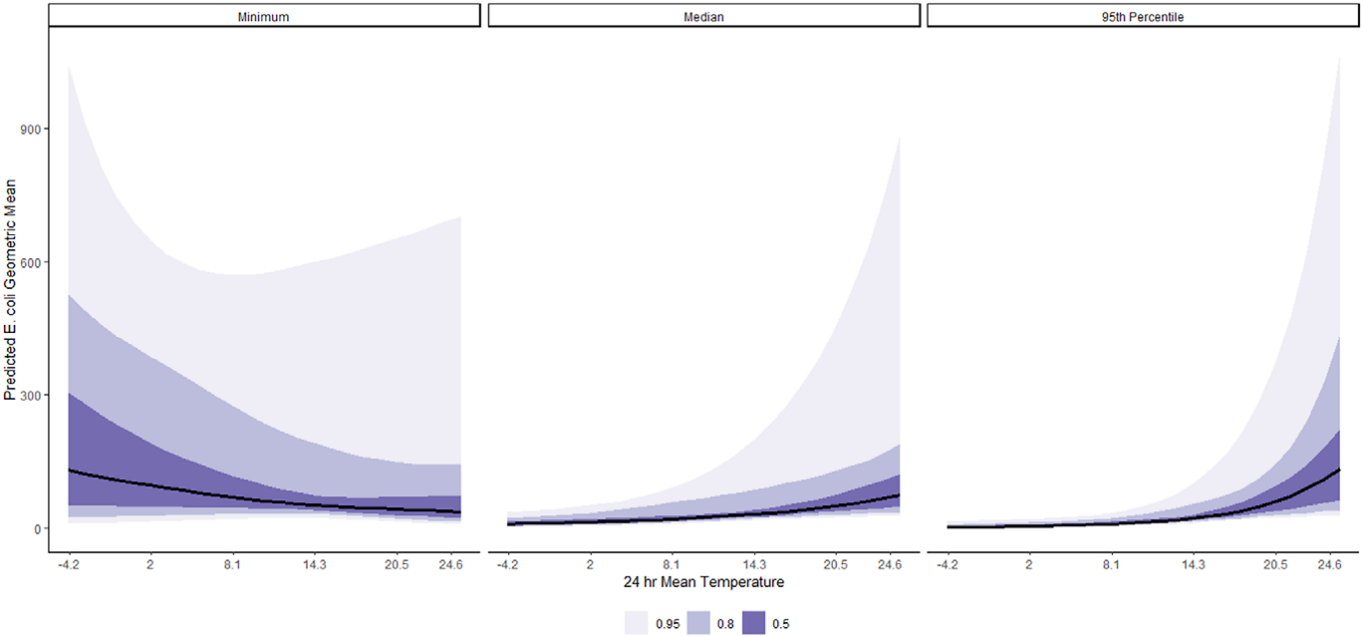
Posterior predictions of the average expected value of the geometric *E. coli* concentration per 24-h mean temperature at minimum (0.01), median (1.29), and 95th percentile (2.13) values of 24-h mean UV index, at beaches in the Metro Vancouver Region, 2013–2021.

Figure 9 (and Figures J to L in the Supplementary Material) shows the posterior predictions for the average expected value of the geometric *E. coli* concentration per value of 24-h air average temperature at different levels (minimum = 0.01, median = 1.29, and 95th percentile = 2.13) of 24-h average UV index, respectively. Conditional on other predictors in the model, on a day with an average value of 24-h mean UV index, a 1-SD (4.1°C) increase in 24-h air mean temperature is associated with a 31% increase in geometric mean *E. coli* counts, on average (Table 1). This association is better illustrated in Figure 9, where an increase in 24-h mean temperature is predicted to increase geometric mean *E. coli* counts at the median or higher values of 24-h mean UV index compared to when the UV index is at its minimum value. This relationship was also notably varied by beaches, as shown in Figures J to L in the Supplementary Material.

In our final model, 4,536 rows (days) were observed entirely, and only 291 rows had at least one missing data for variables in the model (mainly for rainfall with 195 rows; Table D in the Supplementary Material). In the sensitivity analysis of the impact of data missingness, the full Bayesian imputation model converged, and the estimates were similar to those from our models presented in this paper (Table E in the Supplementary Material).

## Discussion

In this study, we used a multilevel, varying slope approach that allowed an in-depth understanding of the environmental predictors of marine water quality via a Bayesian framework that accounts for uncertainty using the estimation of posterior distributions of model parameters. We identified the best-fitting combination of environmental factors to predict *E. coli* concentrations at 15 marine water recreation areas in Metro Vancouver, British Columbia. The yearly average of the geometric mean of *E. coli* varied by area, where higher yearly average values were observed in East False Creek in all study years, with the highest in 2014. Over the years, various factors are considered to influence the higher *E. coli* levels in East False Creek including poor tidal flushing, spring run-off/storm water, boat holding tank releases, and during heavy rainfall releases from combined sewer overflow outfalls [32–34]. News reports also noted the city’s ongoing efforts to reduce the contamination level at this beach [35, 36], including the promotion of a mobile boat sewage pump-out programme [37].

Our mixed-methods analysis showed a positive average effect of the log geometric mean of *E. coli* in the previous sample day on geometric mean *E. coli* concentration, consistent with previous studies elsewhere [38–40]. Even though beach water sampling was not conducted daily in Vancouver, the positive effect of the previous sample day log *E. coli* values on the *E. coli* concentration could indicate the persistence of contamination levels in marine water bodies over several days, conditional on other environmental factors. For example, some *E. coli* strains can survive up to three days in coastal water under normal environmental conditions [16]. Moreover, *E. coli* has a more typical behaviour than *Enterococcus* in attaching to beach sediments of coastal water, which may prolong its survival in such water bodies [41]. Therefore, there is some value in the current public notification approach of using the previous sample day results to make decisions about beach postings. Still, it also ignores the conditional effects of other important environmental factors.

Increased precipitation in the preceding 48 h was found to have a positive average effect on *E. coli* concentration. This association is consistent with previous studies that identified rainfall as a significant predictor variable of marine water quality, which could emanate from faecal pollution in urban run-off and storm water discharge that carry pathogens into marine water bodies [12, 13]. Increased average salinity was found to have an average negative effect on *E. coli* concentration. Some studies have also reported the impact of salinity on microbial marine water quality [14, 38]. The higher salinity levels in water would subject the enteric bacteria to an immediate osmotic up-shock when they entered the coastal water. Therefore, their survival in the marine environment could be primarily influenced by their ability to overcome the up-shock using several osmoregulatory systems [42]. Moreover, some inorganic salts in the water could have toxic effects on the *E. coli* strains [16].

In line with a study on beaches in southern California [43], we found no effect of antecedent dry days on the *E. coli* concentration, which was consistent across all studied beaches. The impact of antecedent dry days could mean that the more extended the days are without rainfall, the more faecal material could accumulate on the beaches’ surrounding areas, which could then be washed into the coastal waters with high concentrations during subsequent rainfall events [44]. However, the survival of *E. coli* in the outside environment could vary and depend on environmental conditions, implying that the increased antecedent dry days decrease the survival rate of the indicator bacteria [45]. Thus, this variable’s lack of an effect in this study could be explained by the destruction of earlier-deposited *E. coli* strains in the surrounding environment in balance with the addition of new faecal material being washed into coastal water bodies [43], provided that the effect of this variable could have also been accounted by other variables (e.g. rainfall, air temperature) in our model.

This study found that increased previous-day mean temperature had a positive average effect on *E. coli* counts at the median or higher UV index values and an average negative effect at the minimum UV index value. Higher temperatures could facilitate *E. coli* survival, growth, and reproduction [46]. The average positive effect of increased temperature on *E. coli* values across the median to higher levels of UV index could be due to the *E. coli* strains that entered the coastal water, as *E. coli* strains from combined sewer overflow outfalls have previously shown a higher survival rate in such an environment compared to *E. coli* strains from sources other than combined sewer overflow outfalls [16]. The *E. coli* strains may experience a starvation adaptation process when they enter the coastal water, following the change to a nutrient-poor environment from a nutrient-rich one [16]. This process would follow by the induction of a protective mechanism against UV radiation stress as a response to starvation stress [47]. This stationary-phase starvation induces specific protein synthesis, enabling the *E. coli* strain to resist higher temperatures [48] and salinity [49].

On the other hand, as no interaction effect of temperature and salinity on *E. coli* strains was reported in prior research [16], the slightly decreasing pattern of *E. coli* concentration with the increasing temperature at the minimum UV index value could be explained by the influence of other factors not included in this analysis. These factors, such as turbidity, the presence of waterfowls, and storm water discharge, influence and interfere or interact with other environmental factors that influence the *E. coli* concentration in marine water bodies [44, 50, 51]. Moreover, *E. coli* strains have a relatively higher inactivation rate with an increase in temperature in marine than freshwater bodies [52]. Studies also indicated the variability in the survival ability between indicator bacteria (e.g. *E. coli* vs. *Enterococci*) [16, 42], thereby suggesting the use of both indicators to comprehensively understand the environmental conditions that affect marine water quality [6, 8].

Overall, this study showed that the effect of the above-discussed predictors of *E. coli* concentration in marine recreational water varied widely by beach. Multiple factors come into effect for the variation shown by beach, with non-point source contamination [1] and altering water dynamics that depend on beach-specific factors considered in this study and other factors not included due to lack of data such as turbidity, the presence of waterfowl, and storm water discharge [44, 50, 51]. Moreover, the beaches adjacent or geographically closely located to East False Creek (with higher *E. coli* levels as described above [32–34]) were more likely to receive contaminated water than beaches situated farther away, thereby contributing to the variation by beach. Thus, the beach-specific slopes for the predictors in our Bayesian mixed-effects modelling would allow us to distinctly understand each predictor’s effect at each beach, thereby reducing the likelihood of reporting crude average effects [53]. We recommend that future studies and predictive models consider whether beach-specific relationships are important and account for these in risk management and communication policies. Still, it is not always the case that such relationships are that different by beach, though they were in this study. For example, in a similar study in Toronto and Niagara, the Niagara beaches were very similar probably due to proximity and most had really excellent water quality, so it might be related to both of those factors and common/different pollution sources [40].

We identified some limitations in this study. First, we excluded potential predictor variables from the final model due to data incompleteness. We extracted data from Fisheries and Oceans Canada for wind speed and direction, gust speed, wave height, and water surface temperature. However, the number of available records was incomplete (i.e. around half of the observations were available per year, linked to our *E. coli* count recorded dates) for all the years except 2016. As a result, these factors were not used. One of the study beaches, Crab Park, was added to the list of beaches monitored by Metro Vancouver in 2014, so there are missing data for that beach in 2013. Second, we were not able to investigate the influence of other environmental variables, such as turbidity, the presence of waterfowl, and storm water discharge, which are also reported to influence *E. coli* concentrations in coastal water [44, 50, 51]. We recommend the collection of these parameters for future beach water quality monitoring to allow trend analysis and to incorporate them and assess the performance of marine recreational water quality prediction models. Third, the weather/environmental variables were taken from nearby stations, so they are proxies for beach-specific measurements. Ideally, we could collect this info separately from each beach to account for local, beach-specific differences.

To conclude, we identified environmental factors associated with marine recreational water quality at 15 beaches in Metro Vancouver, which could inform ongoing beach management strategies and efforts in designing interventions to protect beachgoer health from recreational water illnesses. We identified that days with high rainfall (a 1-SD increase) in the preceding 48 h and high previous-day average air temperature at average or high levels of the previous-day mean UV index resulted in a higher *E. coli* concentration. In contrast, days with high average salinity resulted in lower *E. coli* values in the water bodies. These findings indicate that beach monitoring programmes should be enhanced to address extreme weather events as part of ongoing climate change preparedness efforts. The conditional impact of higher levels of previous sample day *E. coli* concentrations on increasing *E. coli* counts highlights the need for holistic beach water quality management considering the combined effect of the above-mentioned environmental factors and using the previous sample day *E. coli* counts. Finally, we determined that the average effects of the predictor variables on the *E. coli* concentrations varied highly by beaches, which indicates that a beach-specific approach to beach monitoring programmes and predictive models is warranted in Vancouver. Our findings could form the basis for building real-time predictive marine water quality models to enable more timely beach management decision-making. Moreover, the model results could inform potential trends in other marine recreational water settings.

## Supporting information

Desta et al. supplementary material Desta et al. supplementary material

## Data Availability

The environmental data presented in this study are openly available online from the Environment and Climate Change Canada historical data repository from two weather stations (Vancouver Harbour: https://climate.weather.gc.ca/climate_data/daily_data_e.html?hlyRange=1976-01-20%7C2022-09-18&dlyRange=1925-11-01%7C2022-09-17&mlyRange=1925-01-01%7C2007-02-01&StationID=888&Prov=BC&urlExtension=_e.html&searchType=stnProx&optLimit=yearRange&Month=9&Day=12 (accessed on 15 September 2022) and North Vancouver Wharves: https://climate.weather.gc.ca/climate_data/daily_data_e.html?hlyRange=%7C&dlyRange=1962-03-01%7C2023-05-28&mlyRange=1962-01-01%7C2007-02-01&StationID=833&Prov=BC&urlExtension=_e.html&searchType=stnName&optLimit=yearRange&StartYear=1840&EndYear=2023&selRow (accessed on 15 March 2023)), the department of Oceans and Fisheries buoy wave data (https://www.meds-sdmm.dfo-mpo.gc.ca/isdm-gdsi/waves-vagues/data-donnees/data-donnees-eng.asp?medsid=C46131) (accessed on 15 September 2022) and water level data (https://www.isdm-gdsi.gc.ca/isdm-gdsi/twl-mne/inventory-inventaire/data-donnees-eng.asp?user=isdm-gdsi&region=PAC&tst=1&no=7735) (accessed on 15 September 2022), and the NASA database of solar and meteorological parameters (https://power.larc.nasa.gov/data-access-viewer/) (accessed on 15 September 2022). Vancouver’s beach *E. coli* data and all the other extracted data are available from the following GitHub page: https://github.com/bndesta/VancouverRecWater.

## Supplementary material

For supplementary material accompanying this paper visit http://doi.org/10.1017/S0950268824000311. click here to view supplementary material
