# Supplementary material for: Environmental predictors of Escherichia coli concentration at marine beaches in Vancouver, Canada: a Bayesian mixed-effects modelling analysis
Source: Epidemiol Infect. 2024 Feb 26;152:e38. doi: 10.1017/S0950268824000311 (PMC10945941; doi:10.1017/S0950268824000311)
Supplement: Desta et al. supplementary material [file S0950268824000311sup001.docx]

**Supporting information**

for

**Environmental predictors of *Escherichia coli* concentration at Marine beaches in Vancouver, Canada: A Bayesian mixed-effect modelling analysis**

Figure A in S1 Text 2

Figure B in S1 Text 3

Figure C in S1 Text 4

Figure D in S1 Text 5

Figure E in S1 Text 6

Figure F in S1 Text 7

Figure G in S1 Text 8

Figure H in S1 Text 9

Table A in S1 Text 19

Table B in S1 Text 19

Table C in S1 Text 20

Figure I in S1 Text 23

Figure J in S1 Text 24

Figure K in S1 Text 25

Figure L in S1 Text 26

Table D in ST Text 27

Table E in ST Text 28


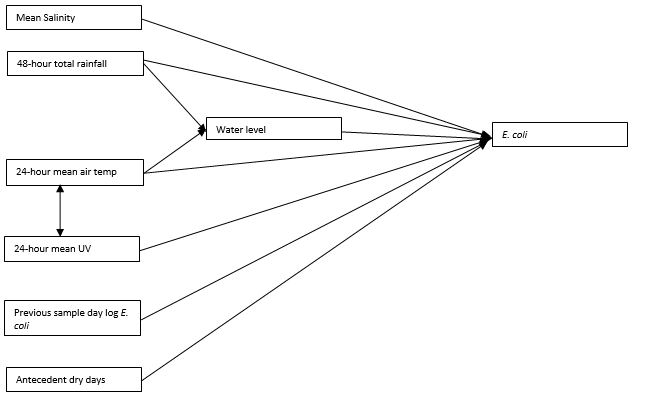


Figure A in S1 Text. Directed Acyclic Graph (DAG) of the relationship between variables affecting E. coli.


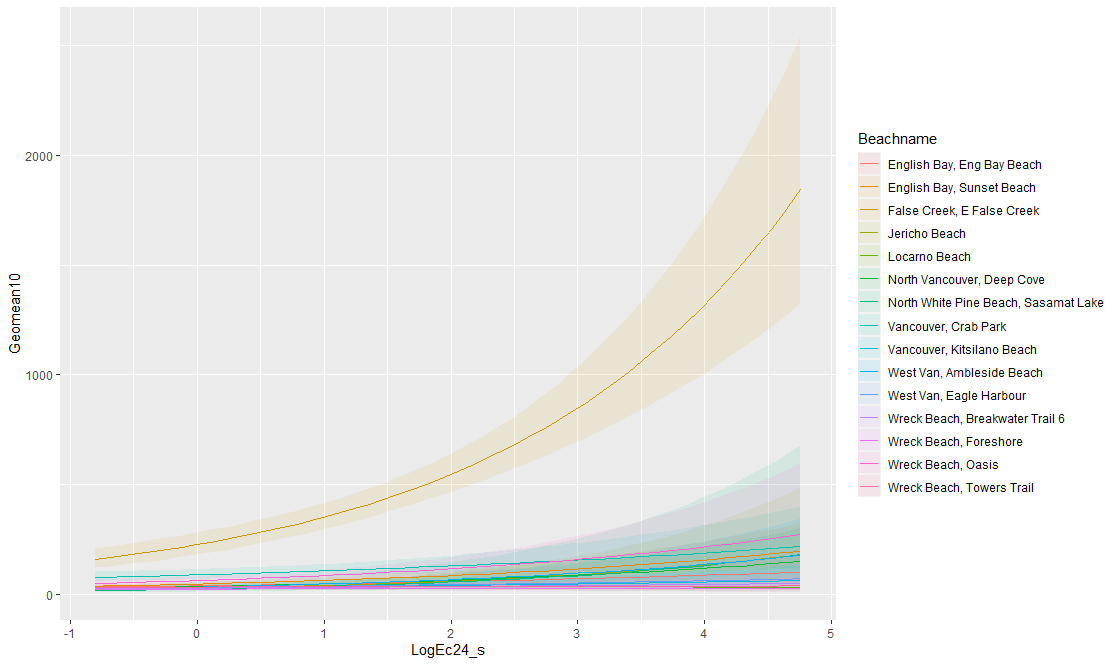


Log Previous Sample Day *E. coli* Geometric Mean

Predicted *E. coli* Geometric Mean

Figure B in S1 Text. Conditional adjusted prediction plots to check the appropriateness of varying slopes for the beach-specific average effect of the previous sample day log geometric mean of *E. coli* on the geometric *E. coli* concentration at beaches in the Metro Vancouver Region, 2013 - 2021.


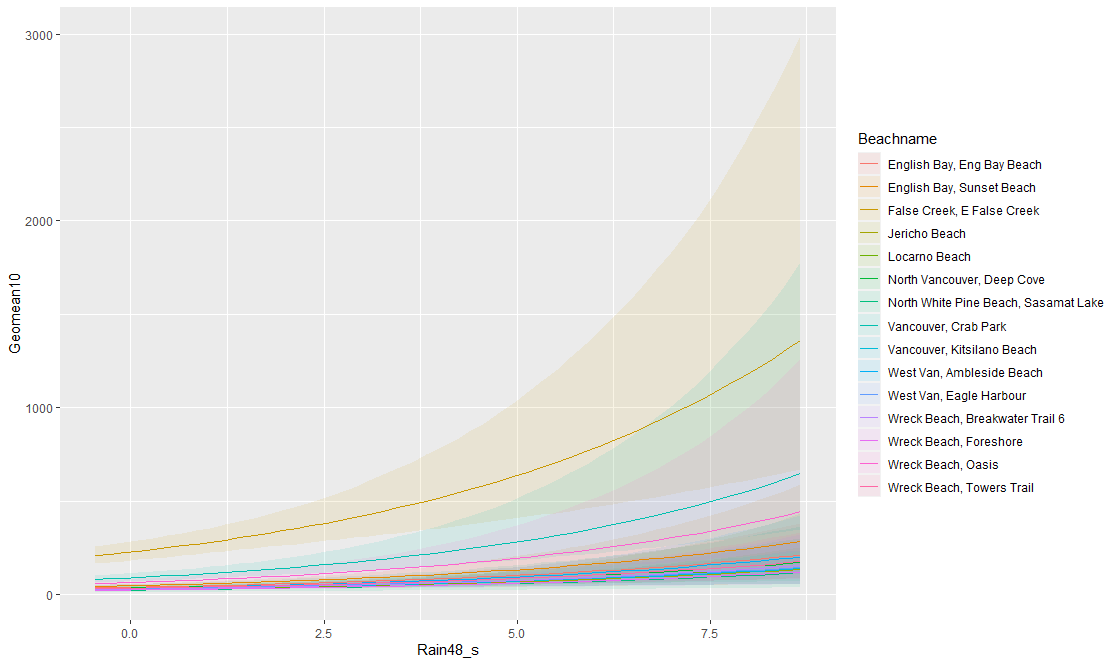


48 hr Rainfall

Predicted *E. coli* Geometric Mean

Figure C in S1 Text. Conditional adjusted prediction plots to check the appropriateness of varying slopes for the beach-specific average effect of 48 h rainfall on the geometric *E. coli* concentration at beaches in the Metro Vancouver Region, 2013 - 2021.


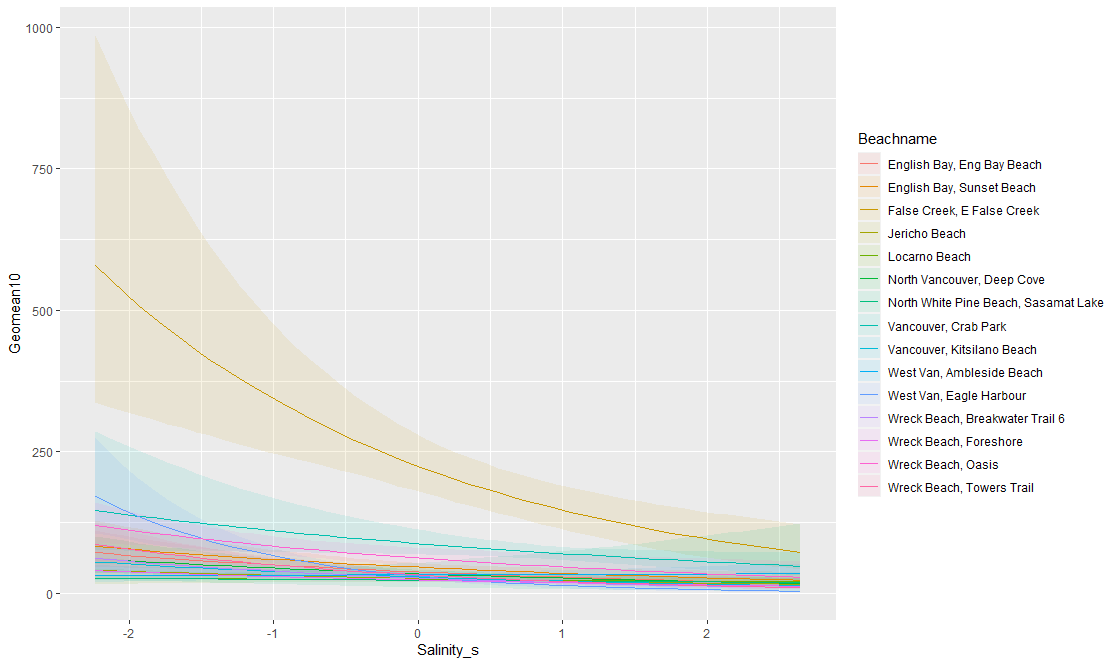


Mean Salinity

Predicted *E. coli* Geometric Mean

Figure D in S1 Text. Conditional adjusted prediction plots to check the appropriateness of varying slopes for the beach-specific average effect of mean salinity on the geometric *E. coli* concentration at beaches in the Metro Vancouver Region, 2013 - 2021.


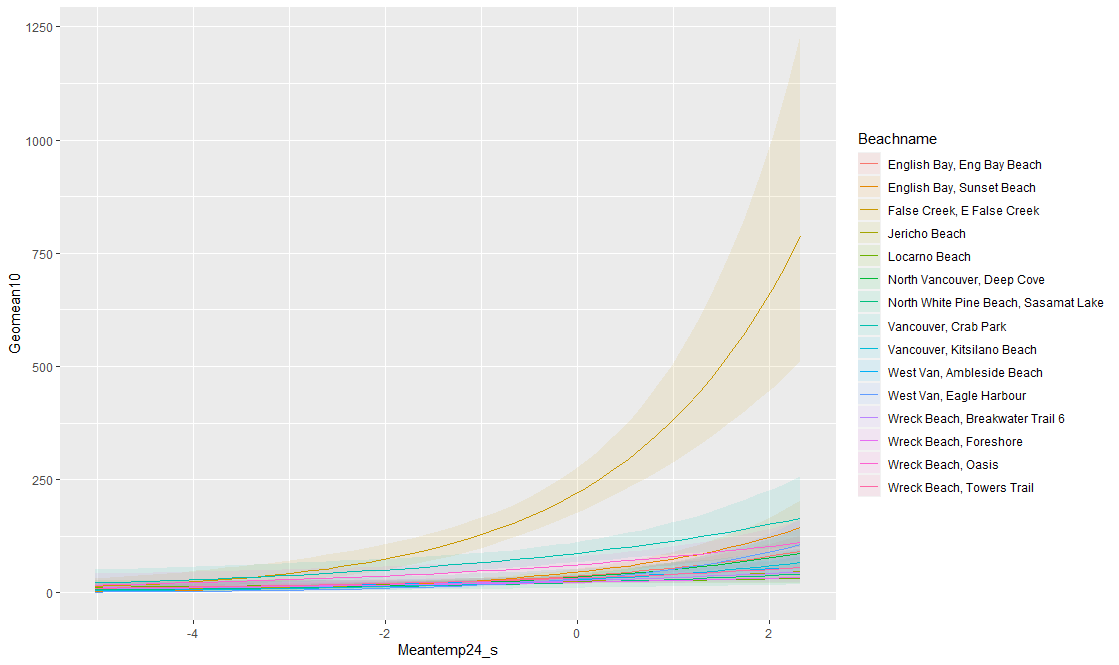


24 hr Mean Temperature

Predicted *E. coli* Geometric Mean

Figure E in S1 Text. Conditional adjusted prediction plots to check the appropriateness of varying slopes for the beach-specific average effect of 24 hr mean temperature on the geometric *E. coli* concentration at beaches in the Metro Vancouver Region, 2013 - 2021.


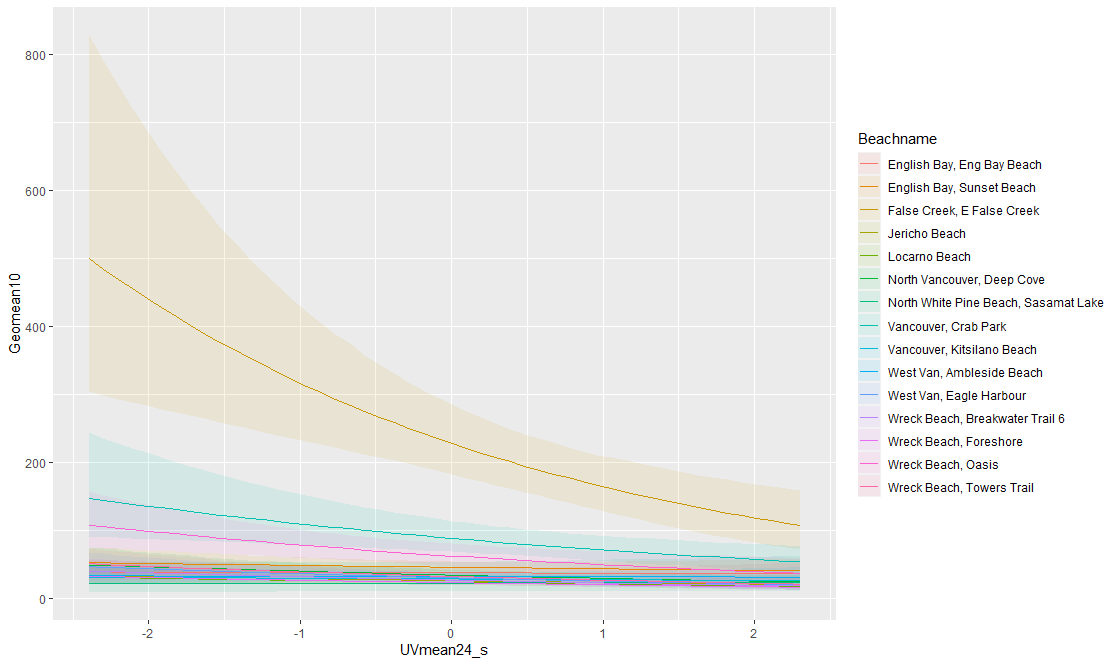


24 hr Mean UV

Predicted *E. coli* Geometric Mean

Figure F in S1 Text. Conditional adjusted prediction plots to check the appropriateness of varying slopes for the beach-specific average effect of 24 hr mean UV index on the geometric *E. coli* concentration at beaches in the Metro Vancouver Region, 2013 - 2021.


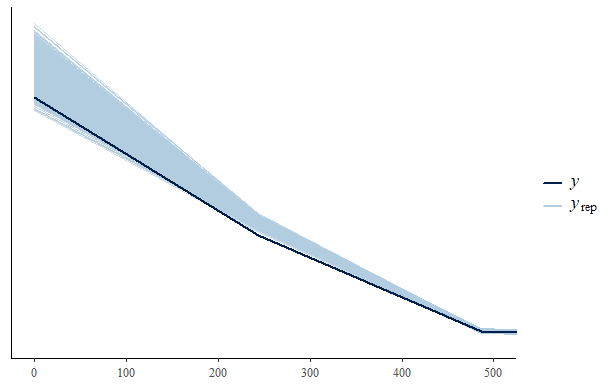


Figure G in S1 Text. Graphical posterior predictive density overlay check showing the observed E. coli values (y) and the kernel density estimate of one of the replications of y from the posterior predictive distribution in our Bayesian mixed-model effects model. (Showing most of the observed data points align with the data we generated in the modelling, indicating that our model accounted for a large proportion of observed values (y)).


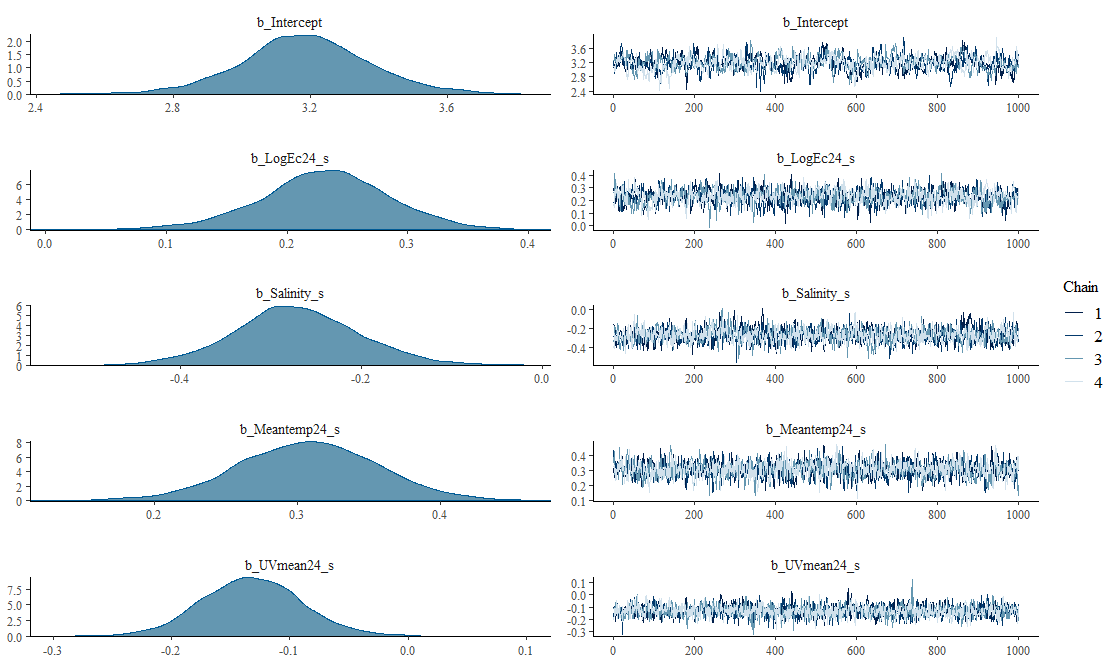


Figure H in S1 Text. Markov chain Monte Carlo (MCMC) trace plot showing convergence in the final model. (Model convergence can be detected when the chains explore the same region of each parameter value)


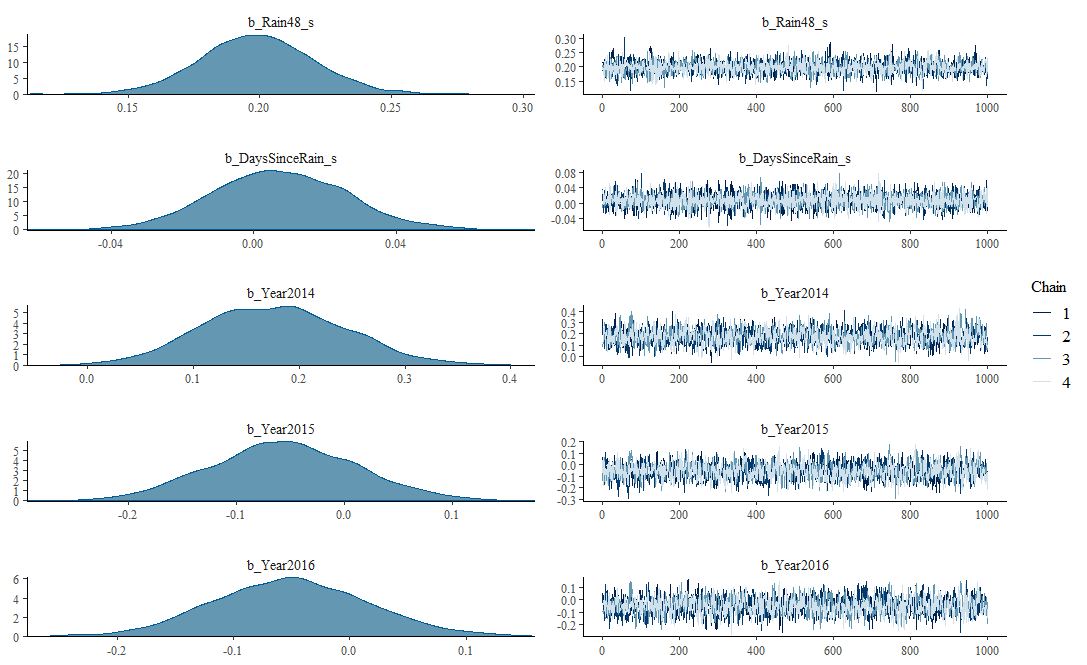


Figure H in S1 Text (continued). Markov chain Monte Carlo (MCMC) trace plot showing convergence in the final model. (Model convergence can be detected when the chains explore the same region of each parameter value)


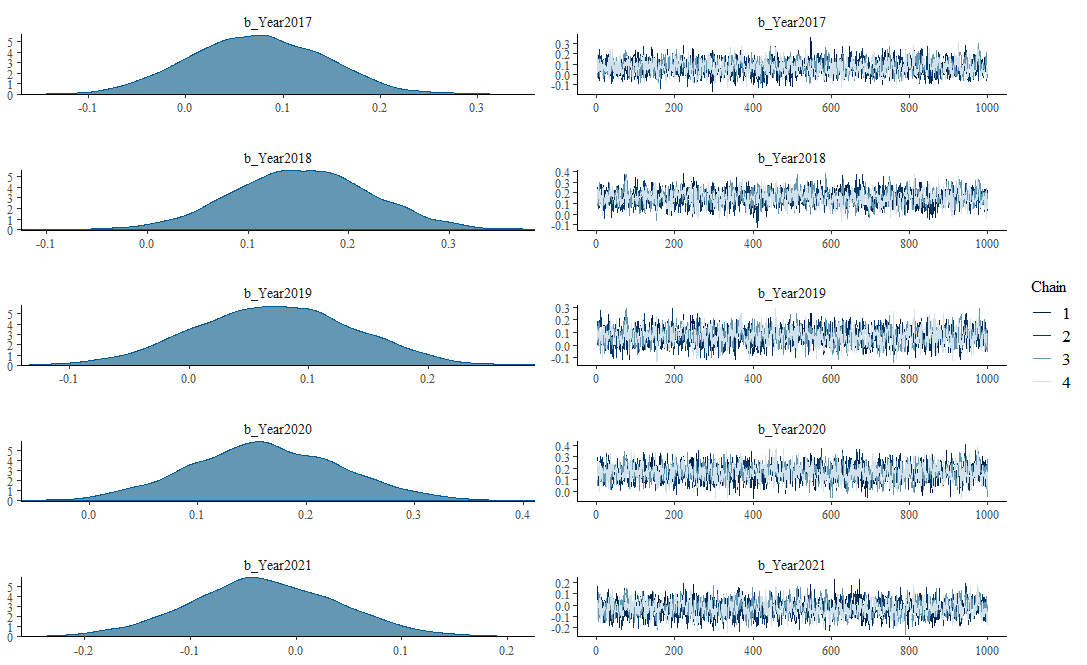


Figure H in S1 Text (continued). Markov chain Monte Carlo (MCMC) trace plot showing convergence in the final model. (Model convergence can be detected when the chains explore the same region of each parameter value)


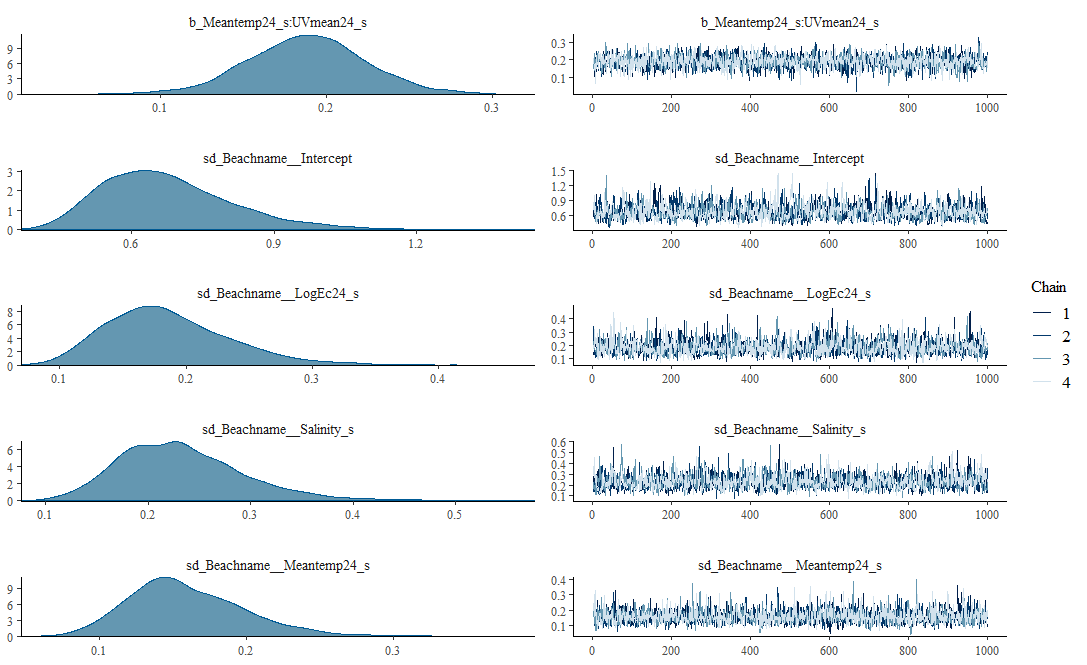


Figure H in S1 Text (continued). Markov chain Monte Carlo (MCMC) trace plot showing convergence in the final model. (Model convergence can be detected when the chains explore the same region of each parameter value)


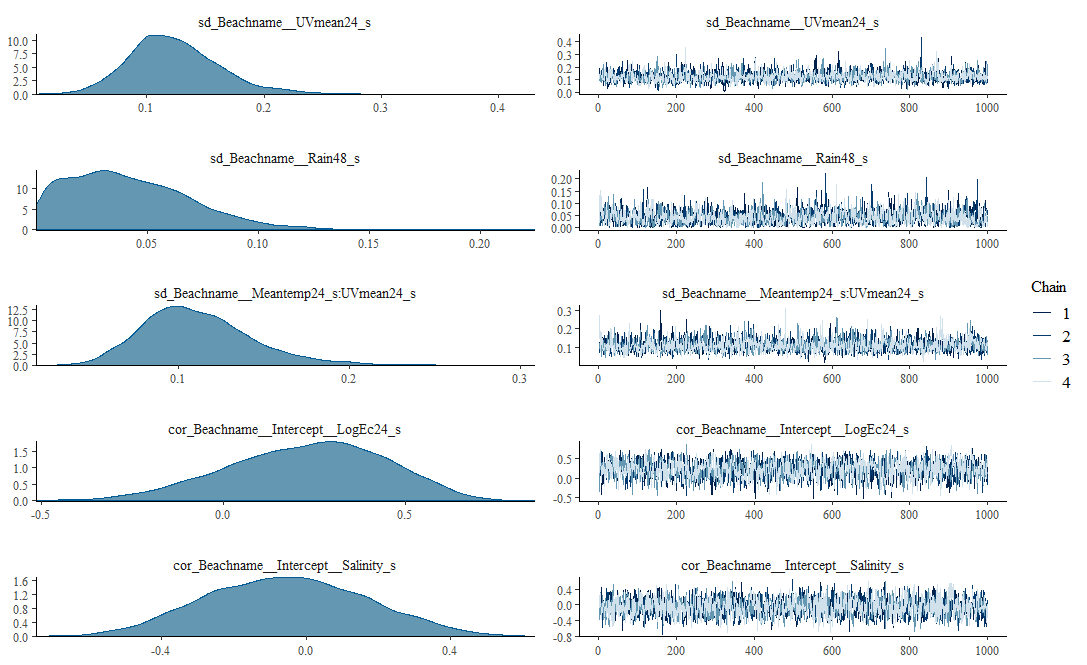


Figure H in S1 Text (continued). Markov chain Monte Carlo (MCMC) trace plot showing convergence in the final model. (Model convergence can be detected when the chains explore the same region of each parameter value)


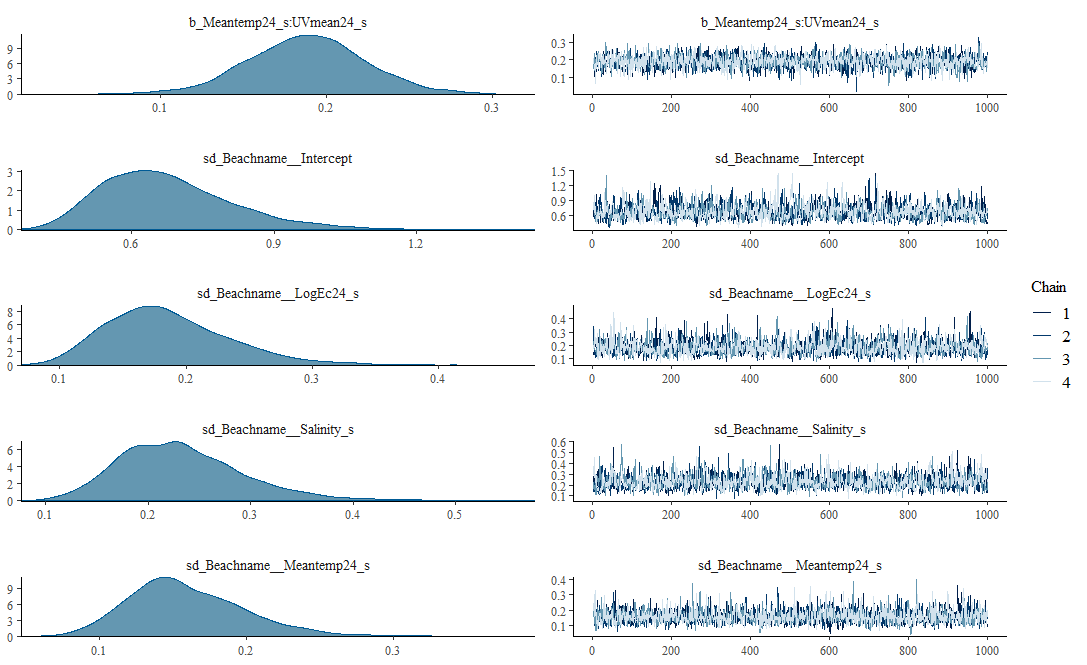


Figure H in S1 Text (continued). Markov chain Monte Carlo (MCMC) trace plot showing convergence in the final model. (Model convergence can be detected when the chains explore the same region of each parameter value)


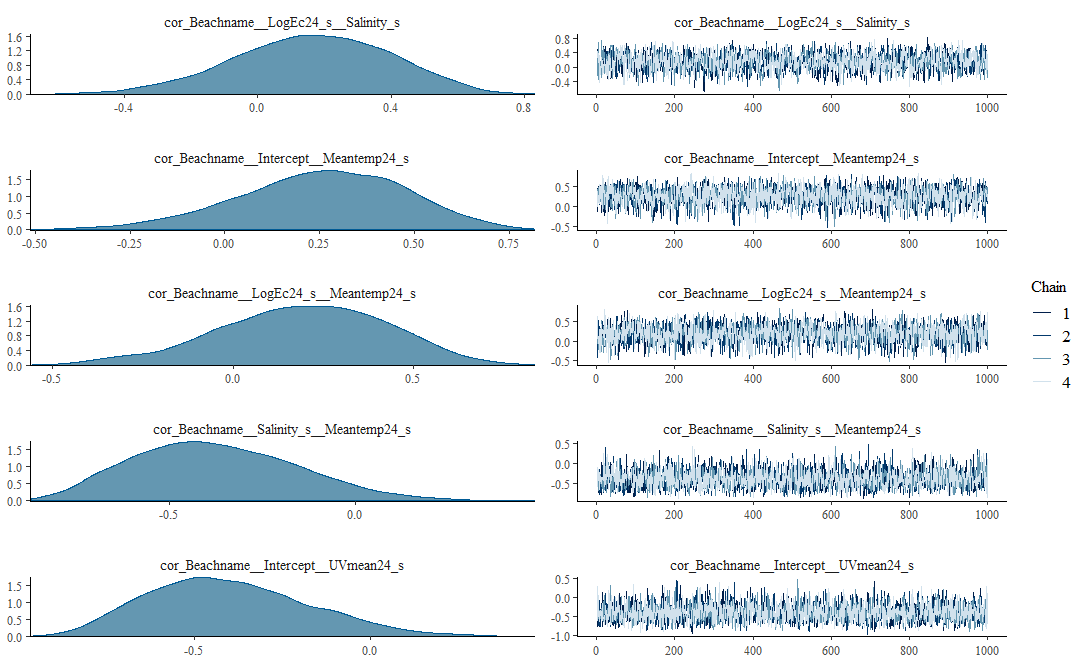


Figure H in S1 Text (continued). Markov chain Monte Carlo (MCMC) trace plot showing convergence in the final model. (Model convergence can be detected when the chains explore the same region of each parameter value)


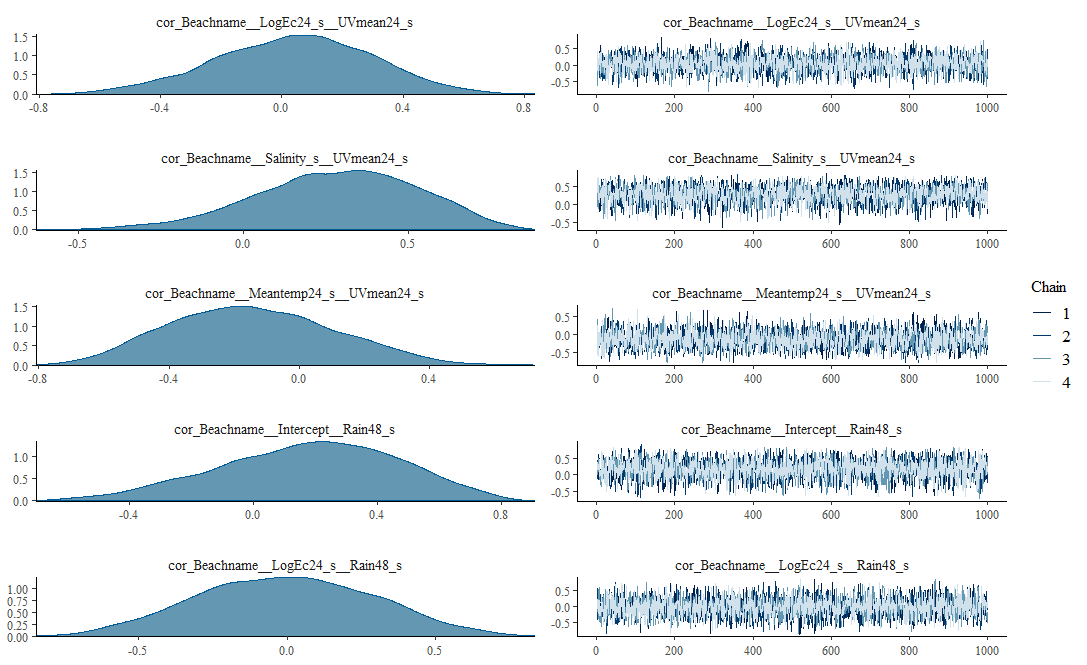


Figure H in S1 Text (continued). Markov chain Monte Carlo (MCMC) trace plot showing convergence in the final model. (Model convergence can be detected when the chains explore the same region of each parameter value)


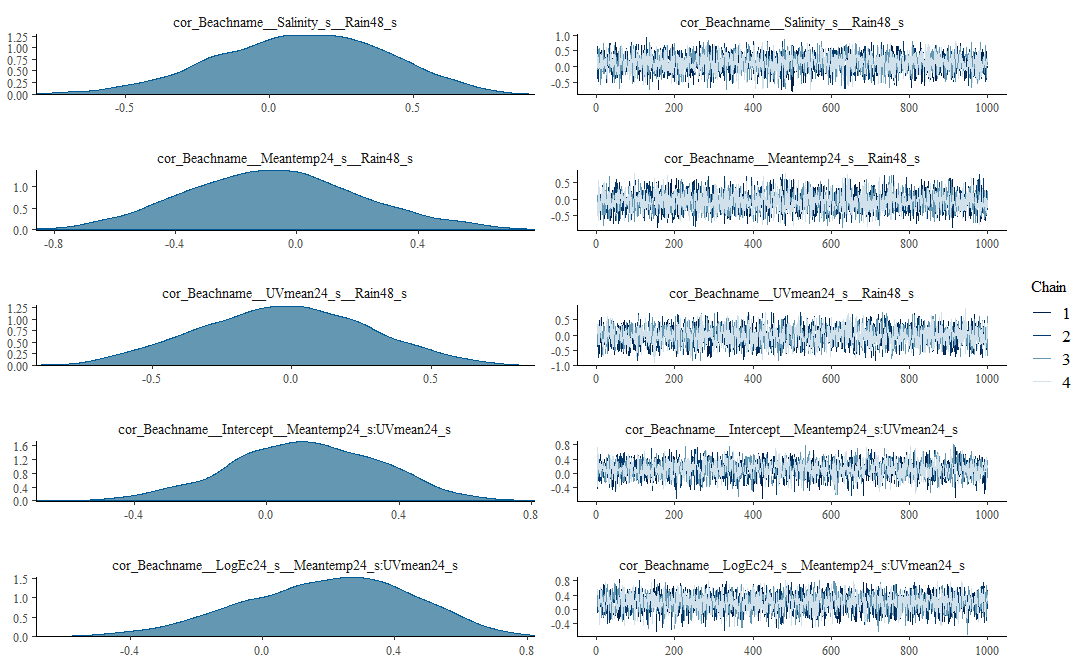


Figure H in S1 Text (continued). Markov chain Monte Carlo (MCMC) trace plot showing convergence in the final model. (Model convergence can be detected when the chains explore the same region of each parameter value)


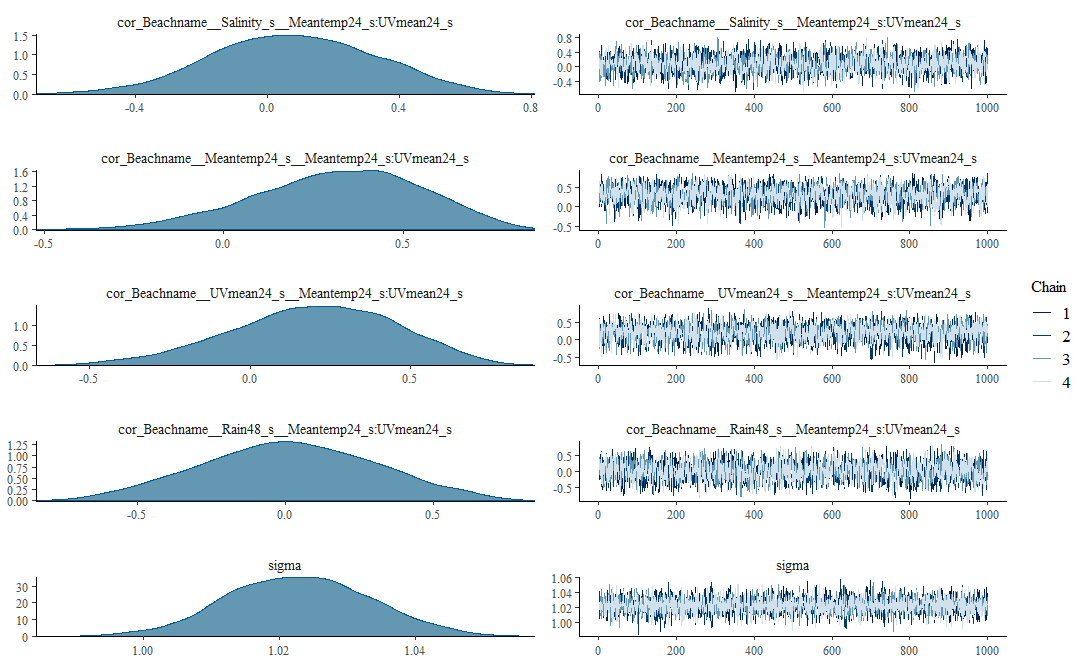


Figure H in S1 Text (continued). Markov chain Monte Carlo (MCMC) trace plot showing convergence in the final model. (Model convergence can be detected when the chains explore the same region of each parameter value)

Table A in ST Text. Showing Loo model comparison using expected log predictive density difference. The final model (top row with zero value) is better.

| Model comparison | Expected log predictive density difference | Standard error difference |
| --- | --- | --- |
| Model containing varying slope predictors, beach a varying effect and a two-way interaction effect | 0.0 | 0.0 |
| Model containing beach as a varying intercept effect | -103.9 | 16.3 |

Table B in ST Text. Showing Loo model comparison using expected log predictive density difference. The final model (top row with zero value) is better.

| Model comparison | Expected log predictive density difference | Standard error difference |
| --- | --- | --- |
| Model not containing varying slope for antecedent dry days | 0.0 | 0.0 |
| Model containing varying slope for antecedent dry days | -0.7 | 0.4 |

Table C in ST Text. Bayesian log-normal mixed-effects model of the relationship between environmental factors and geometric mean *E. coli* concentration at 15 beaches in the Metro Vancouver Region, 2013-2021 (Group-effect parameters).

| Outcome/parameter^a^ | | Estimate^b^ | 95% credible interval | R-hat^c^ | Bulk ESS^c^ | Tail ESS^c^ |
| --- | --- | --- | --- | --- | --- | --- |
| **Group-level effects** | | | | | | |
| SD (Intercept) | | 0.68 | (0.46, 1.01) | 1 | 1211 | 1949 |
| SD (Previous sample day log *E. coli*) | | 0.19 | (0.11, 0.31) | 1 | 1760 | 2648 |
| SD (48 hr total rainfall) | | 0.23 | (0.13, 0.38) | 1 | 1546 | 2069 |
| SD (Mean Salinity) | | 0.16 | (0.09, 0.25) | 1 | 2650 | 2850 |
| SD (Antecedent dry days) | | 0.12 | (0.06, 0.21) | 1 | 2003 | 2243 |
| SD (24 hr mean air temperature) | | 0.04 | (0.00, 0.11) | 1 | 1449 | 1984 |
| SD (24 hr mean UV) | | 0.11 | (0.06, 0.19) | 1 | 2153 | 2812 |
| SD (24 hr mean air temperature * 24 hr mean UV) | | 0.23 | (-0.22, 0.61) | 1 | 2127 | 3141 |
| COR (Intercept, Previous sample day log *E. coli*) | | -0.05 | (-0.47, 0.37) | 1 | 2296 | 2632 |
| COR (Intercept, Mean Salinity) | | 0.16 | (-0.32, 0.59) | 1 | 1656 | 2557 |
| COR (Previous sample day log *E. coli*, Mean Salinity) | | 0.25 | (-0.21, 0.65) | 1 | 2565 | 2798 |
| COR (Intercept, 24 hr mean air temperature) | | 0.19 | (-0.31, 0.62) | 1 | 2409 | 2951 |
| COR (Previous sample day log_10_ *E. coli*, 24 hr mean air temperature) | | -0.37 | (-0.75, 0.09) | 1 | 2747 | 3235 |
| COR (Mean Salinity, 24 hr mean air temperature) | | -0.40 | (-0.78, 0.08) | 1 | 3353 | 3027 |
| COR (Intercept, 24 hr mean UV) | | 0.04 | (-0.49, 0.53) | 1 | 3272 | 2426 |
| COR (Previous sample day log *E. coli*, 24 hr mean UV) | | 0.28 | (-0.23, 0.71) | 1 | 3145 | 3125 |
| COR (Mean Salinity, 24 hr mean UV) | | -0.14 | (-0.60, 0.36) | 1 | 2827 | 2555 |
| COR (24 hr mean air temperature, 24 hr mean UV) | | 0.18 | (-0.41, 0.69) | 1 | 4615 | 2828 |
| COR (Intercept, 48 hr total rainfall) | | -0.01 | (-0.57, 0.57) | 1 | 4767 | 3206 |
| COR (Previous sample day log *E. coli*, 48 hr total rainfall) | | 0.11 | (-0.49, 0.65) | 1 | 4433 | 3387 |
| COR (Mean Salinity, 48 hr total rainfall) | | -0.08 | (0.62, 0.52) | 1 | 4822 | 3191 |
| COR (24 hr mean air temperature, 48 hr total rainfall) | | -0.03 | (-0.61, 0.54) | 1 | 3942 | 3389 |
| COR (Intercept, 24 hr mean air temperature * 24 hr mean UV) | | 0.12 | (-0.34, 0.54) | 1 | 3137 | 2512 |
| COR (Previous sample day log *E. coli*, 24 hr mean air temperature * 24 hr mean UV) | | 0.20 | (-0.32, 0.64) | 1 | 3510 | 3155 |
| COR (Mean Salinity, 24 hr mean air temperature * 24 hr mean UV) | | 0.08 | (-0.42, 0.56) | 1 | 3304 | 3277 |
| COR (24 hr mean air temperature, 24 hr mean air temperature * 24 hr mean UV) | | 0.32 | (-0.17, 0.73) | 1 | 3022 | 2944 |
| COR (24 hr mean UV, 24 hr mean air temperature * 24 hr mean UV) | | 0.20 | (-0.34, 0.66) | 1 | 2982 | 3386 |
| COR (48 hr total rainfall, 24 hr mean air temperature * 24 hr mean UV) | | 0.01 | (-0.56, 0.59) | 1 | 2454 | 3126 |
| **Fixed effects** | | | | | | |
| Year | 2013 | Ref. | Ref. | Ref. | Ref. | Ref. |
|  | 2014 | 0.18 | (0.05, 0.13) | 1 | 1735 | 2683 |
|  | 2015 | -0.06 | (-0.19, 0.08) | 1 | 1629 | 2214 |
|  | 2016 | -0.05 | (-0.18, 0.08) | 1 | 1691 | 2543 |
|  | 2017 | 0.07 | (-0.06, 0.21) | 1 | 1718 | 2447 |
|  | 2018 | 0.15 | (0.02, 0.29) | 1 | 1609 | 2211 |
|  | 2019 | 0.07 | (-0.06, 0.20) | 1 | 1785 | 2579 |
|  | 2020 | 0.16 | (0.03, 0.30) | 1 | 1924 | 2788 |
|  | 2021 | -0.03 | (-0.17, 0.11) | 1 | 1721 | 2859 |


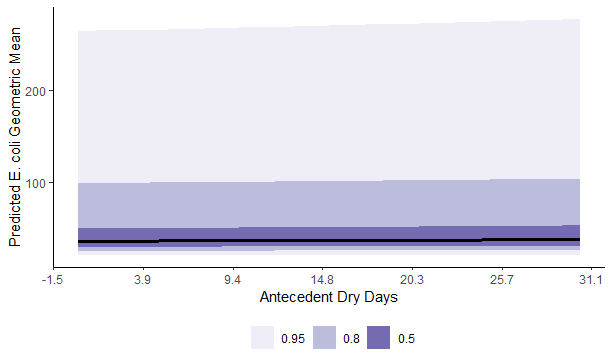


Figure I in S1 Text. Posterior predictions of the average expected value of the geometric *E. coli* concentration per values of antecedent dry days at beaches in the Metro Vancouver Region, 2013 - 2021.


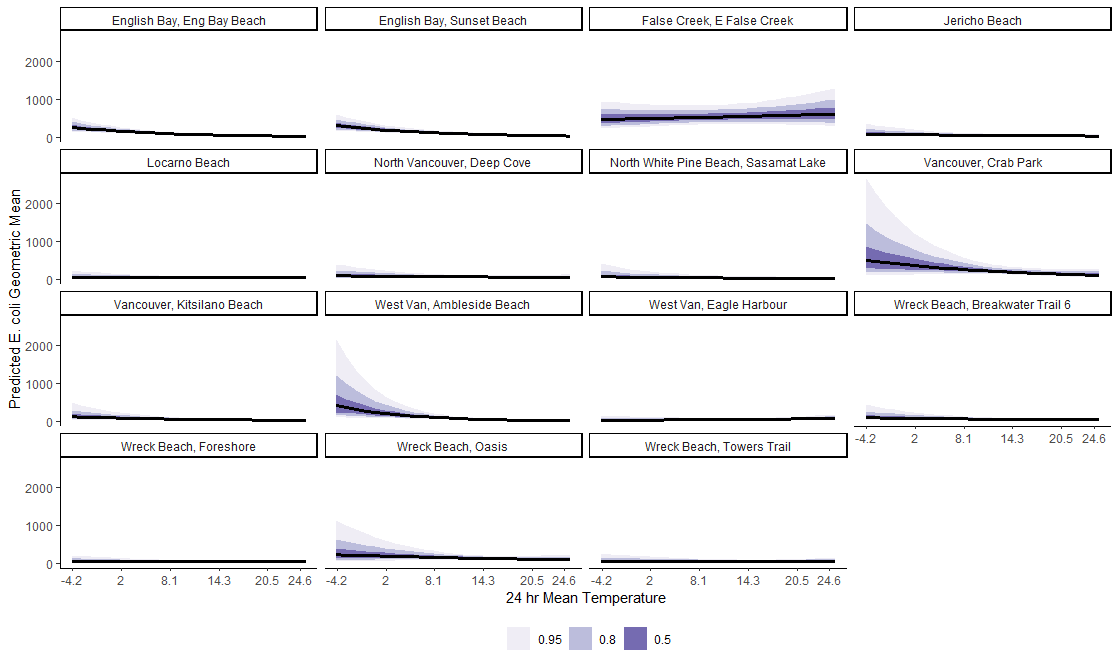


Figure J in S1 Text. Posterior predictions of the beach-specific average expected value of the geometric *E. coli* concentration per 24 hr mean temperature at minimum (0.01) value of 24 hr mean UV index, at beaches in the Metro Vancouver Region, 2013 - 2021.


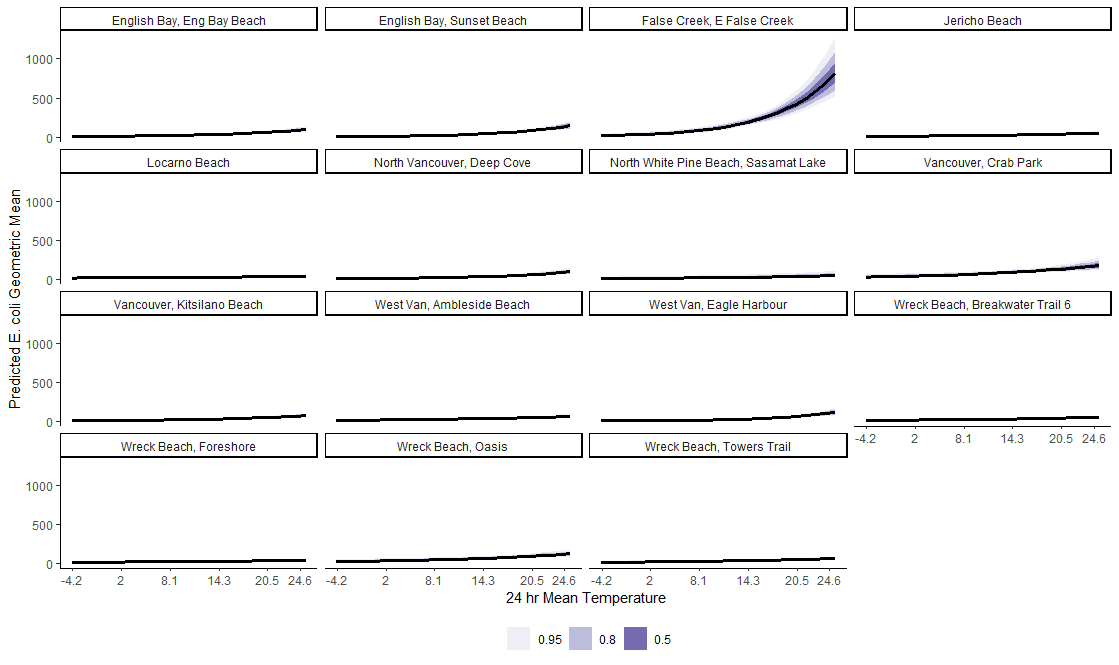


Figure K in S1 Text. Posterior predictions of the beach-specific average expected value of the geometric *E. coli* concentration per 24 hr mean temperature at median (1.29) value of 24 hr mean UV index, at beaches in the Metro Vancouver Region, 2013 - 2021.


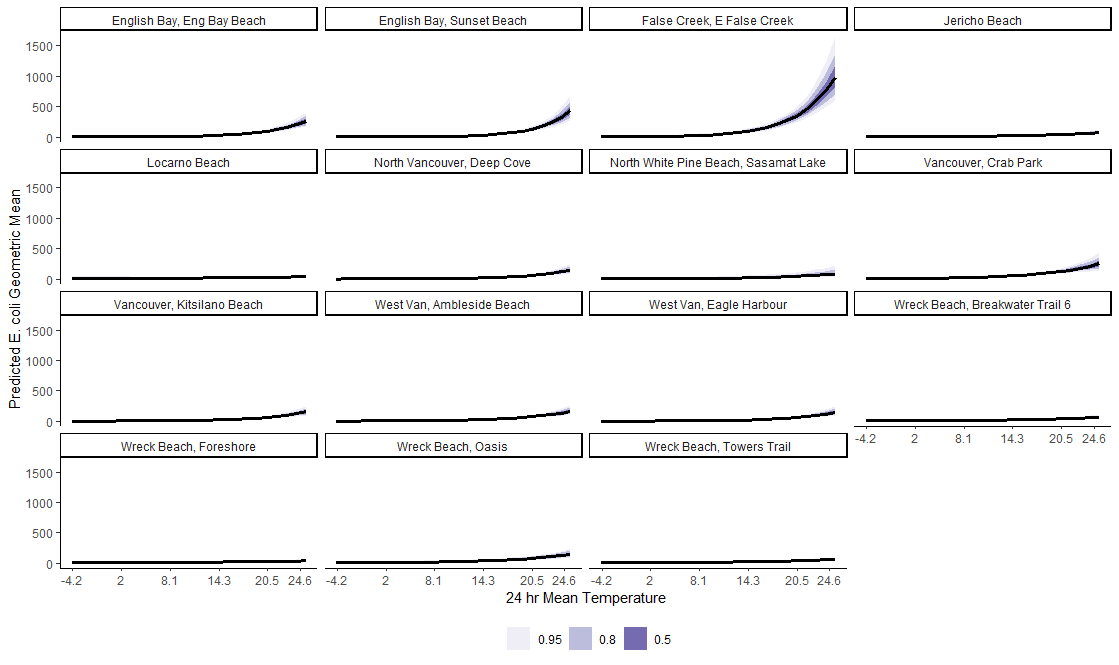


Figure L in S1 Text. Posterior predictions of the beach-specific average expected value of the geometric *E. coli* concentration per 24 hr mean temperature at 95^th^ percentile (2.13) value of 24 hr mean UV index, at beaches in the Metro Vancouver Region, 2013 - 2021.

Sensitivity Analysis

Table D in ST Text. Data missingness in among the variables used in the final Bayesian log-normal mixed-effects model of the relationship between environmental factors and geometric mean *E. coli* concentration at 15 beaches in the Metro Vancouver Region, 2013-2021.

|  | 24 hr mean UV | Antecedent dry days | Year | Beach Name | Date | Geometric mean of *E. coli* | Mean Salinity | Previous sample day log *E. coli* geomean | 24 hr mean air temperature | 48 hr total rainfall | Total |
| --- | --- | --- | --- | --- | --- | --- | --- | --- | --- | --- | --- |
| 4536 | 1 | 1 | 1 | 1 | 1 | 1 | 1 | 1 | 1 | 1 | 0 |
| 145 | 1 | 1 | 1 | 1 | 1 | 1 | 1 | 1 | 1 | 0 | 1 |
| 50 | 1 | 1 | 1 | 1 | 1 | 1 | 1 | 1 | 0 | 0 | 2 |
| 22 | 1 | 1 | 1 | 1 | 1 | 1 | 1 | 0 | 1 | 1 | 1 |
| 17 | 1 | 1 | 1 | 1 | 1 | 1 | 0 | 1 | 1 | 1 | 1 |
| 7 | 1 | 1 | 1 | 1 | 1 | 0 | 1 | 1 | 1 | 1 | 1 |
| Total | 0 | 0 | 0 | 0 | 0 | 7 | 17 | 22 | 50 | 195 | 291 |

Table E in ST Text. Comparison of model outputs from the final Bayesian log-normal mixed-effects model without treating missing observation and with treating missing information with full Bayesian imputation, of the relationship between environmental factors and geometric mean *E. coli* concentration at 15 beaches in the Metro Vancouver Region, 2013-2021.

|  | Final model with out treating missing data | | | Final model treating missing data using full Bayesian imputation | | |
| --- | --- | --- | --- | --- | --- | --- |
| Outcome/parameter^a^ | Estimate^b^ | Standard error | 95% credible interval | Estimate^b^ | Standard error | 95% credible interval |
| **Fixed-level effects** | | | | | | |
| Intercept | 3.17 | 0.19 | (2.79, 3.54) | 3.17 | 0.18 | (2.81, 3.51) |
| Previous sample day log *E. coli* geomean | 0.23 | 0.05 | (0.13, 0.34) | 0.23 | 0.05 | (0.12, 0.33) |
| 48 hr total rainfall | 0.20 | 0.02 | (0.16, 0.24) | 0.19 | 0.02 | (0.15, 0.24) |
| Mean Salinity | -0.28 | 0.07 | (-0.41, -0.13) | -0.28 | 0.07 | (-0.42, -0.15) |
| Antecedent dry days | 0.01 | 0.02 | (-0.03, 0.04) | 0.01 | 0.02 | (-0.03, 0.04) |
| 24 hr mean air temperature | 0.31 | 0.05 | (0.21, 0.41) | 0.31 | 0.05 | (0.21, 0.41) |
| 24 hr mean UV | -0.13 | 0.04 | (-0.22, -0.05) | -0.14 | 0.04 | (-0.23, -0.05) |
| 24 hr mean air temperature * 24 hr mean UV (*interaction term*) | 0.19 | 0.04 | (0.11, 0.26) | 0.18 | 0.04 | (0.10, 0.25) |
| **Group-level effects** for Beach (SD) | 1.02 | 0.01 | (1.00, 1.04) | 1.02 | 0.01 | (1.00, 1.04) |

^a^ Models conditioned on study year as a fixed effect

^b^ All the fixed-effect estimates and credible intervals are shown here on the mean centered and standardized scale.
